# From detection to decision: molecular, genomic, and AI innovations for managing *Ganoderma boninense* in oil palm

**DOI:** 10.1080/21645698.2026.2756004

**Published:** 2026-11-06

**Authors:** Qudrat Ullah, Waqas Haider, Farhang Hameed Awlqadr, Ahmed Bilal, Muhammad Tayyab Arshad, Uzma Faridi, Ahmadullah Zahir

**Affiliations:** a Food Technology and Innovation Research Center of Excellence, Department of Agro-Industry, School of Agricultural Technology and Food Industry, Walailak University, Thasala, Nakhon Si Thammarat, Thailand; b Food Science and Quality Control, Halabja Technical College, Sulaimani Polytechnic University, Sulaymaniyah, Iraq; c University Institute of Diet and Nutritional Sciences, the University of Lahore, Lahore, Pakistan; d Functional Food and Nutrition Program, Prince of Songkla University, Hat Yai, Thailand; e Department of Biochemistry, Faculty of Science, University of Tabuk, Tabuk, Saudi Arabia; f Faculty of Veterinary Sciences, Department of Food Science and Technology, Afghanistan National Agricultural Sciences and Technology University, Kandahar, Afghanistan

**Keywords:** Artificial intelligence, basal stem rot, CRISPR, *Ganoderma boninense*, molecular diagnostics, oil palm, remote sensing

## Abstract

*Ganoderma boninense* causes basal stem rot (BSR) in oil palm, resulting in substantial economic losses, particularly for smallholders who contribute approximately 40% of global production. This review synthesizes advances in molecular diagnostic techniques (qPCR, LAMP), whole-genome sequencing of resistant *Elaeis guineensis* genotypes, and AI-driven remote sensing to enhance understanding of *G. boninense* pathobiology. Soilborne mycelia of the pathogen can persist for more than 10 y. Asymptomatic infections, which are frequently missed by conventional methods, can lead to significant disease spread within plantations over 2 y. CRISPR-based paper-strip tests have demonstrated high sensitivity (detecting < 1 pg DNA in laboratory settings), while marker-assisted selection has helped identify additional resistance QTLs. Trichoderma biofilms and biodegradable fumigants reduced soil inoculum by 50–75% in controlled studies. AI-based decision support systems have demonstrated promising accuracy for guiding interventions and potential cost savings, although further field validation across diverse conditions is needed.

## 1. Introduction

Oil palm (*Elaeis guineensis*) is a cornerstone of global agriculture, underpinning food, fuel, cosmetic, and industrial supply chains, with an estimated market value exceeding 80 billion USD annually.^1^ It is predominantly cultivated in Southeast Asia, where Malaysia and Indonesia account for over 85% of global production. Oil palm also thrives in Africa and Latin America, supporting rural livelihoods and contributing significantly to national Gross Domestic Product (GDPs).^2^ In Malaysia alone, oil palm plantations employ over 500,000 workers and contribute 4–5% to the country’s GDP, underscoring their significant socioeconomic importance.^3^ However, the sustainability of this vital crop is increasingly threatened by BSR, a devastating disease caused by the white-rot fungus *G. boninense*, which poses severe economic and ecological challenges.^4^ This soil-borne pathogen infects roots and degrades vascular tissues, leading to wilting, decay, and premature plant death.^5^ *G. boninense* also targets other economically important crops such as coconut (*Cocos nucifera*) and rubber (*Hevea brasiliensis*), thus its agricultural impact is exacerbated.^6^ Its symptoms include, but are not limited to, frond yellowing, stem base decay, and fruiting body formation; however, early stages are asymptomatic and thus difficult to identify in time. In heavily impacted plantations, BSR can reduce the output by up to 80%, undermine long-term productivity and require massive replanting.^7^

Annual global economic losses from BSR exceed tens of billions USD due to yield decline, premature felling, and replanting, with smallholders (40% of production) particularly vulnerable.^8^ In Malaysia and Indonesia, plantations report 10–30% productivity losses on infested sites, with the possibility of total crop failure over 5–10 years of infection in some estates.^9^ Ecologically, BSR contributes by disturbing the health of the soil and the ecosystem services. The soil organic carbon (SOC) structure continues to be degraded, the diversity of microbes is reduced, and carbon sequestration is impaired due to incessant infections and falls, which compromises biodiversity in tropical agroecosystems.^10^ These cumulative effects indicate the necessity to employ viable ways of managing *G. boninense*. The task of controlling BSR is full of difficulties. Early infections are extremely hard to diagnose because they are asymptomatic and mostly appear once it is too late and permanent damage has been done. Persistence of the pathogen in soil, assisted by robust *basidiospores* and *chlamydospores*, allows long-term survival and makes its eradication almost impossible.^11^ The current treatments (including fungicides such as hexaconazole), as well as cultural means, such as sanitation or soil mounding, are labor-intensive, inconsistently effective, and environmentally concerning. In addition, the lack of genetically resistant oil palm varieties hampers breeding approaches, and field-based diagnosis detached from using visual symptoms or morphological characterization is not useful in massive plantations.^12^ Standard detection strategies, including symptom assessments and PCR have limited capacity in proving the necessity for advanced diagnostics as these approaches tend to struggle with picking up early signs of infection and are often too difficult to be used in the field. There is an urgent need for fast, non-invasive, and large-scale tools, for example, biosensors, remote imaging, AI-driven analytics, and biomarker assays, that allow prompt detection and rendering action.^13^ Detailed and comprehensive study of the literature suggests that sustainable management of *G. boninense* can be achieved employing integrated strategies including biocontrol agents, pesticides, and resistance traits of the host plant.^14^ This review aims to provide a comprehensive, critical synthesis of recent advances in the management of *Ganoderma boninense*-induced basal stem rot (BSR) in oil palm. Specifically, it examines molecular and genomic tools for early detection and resistance breeding, AI-powered remote sensing and decision-support systems for precision intervention, and integrated management strategies suitable for both large plantations and smallholders. By structuring the analysis around a “Detection-to-Decision” framework and evaluating the readiness, limitations, and synergies of these technologies under Southeast Asian field conditions, this review seeks to bridge the gap between laboratory innovations and practical, scalable solutions for sustainable BSR management.

## 2. Occurrence of BSR and Management Strategies

Basal stem rot (BSR) caused by *Ganoderma boninense* is most prevalent in Southeast Asia, particularly in Malaysia and Indonesia, the world’s leading oil palm producers. Significant outbreaks have also been reported in parts of Africa (e.g., Nigeria and Cameroon) and Latin America (e.g., Brazil and Colombia), though the scale and impact are generally lower than in Southeast Asia.^4,15,16^

Figure 1 provides a global overview of BSR distribution alongside major scientific tools developed for its management. This visualization highlights the concentration of the disease in major production regions and synthesizes key diagnostic, host defense, and control approaches reported in the literature.

**Figure 1.**
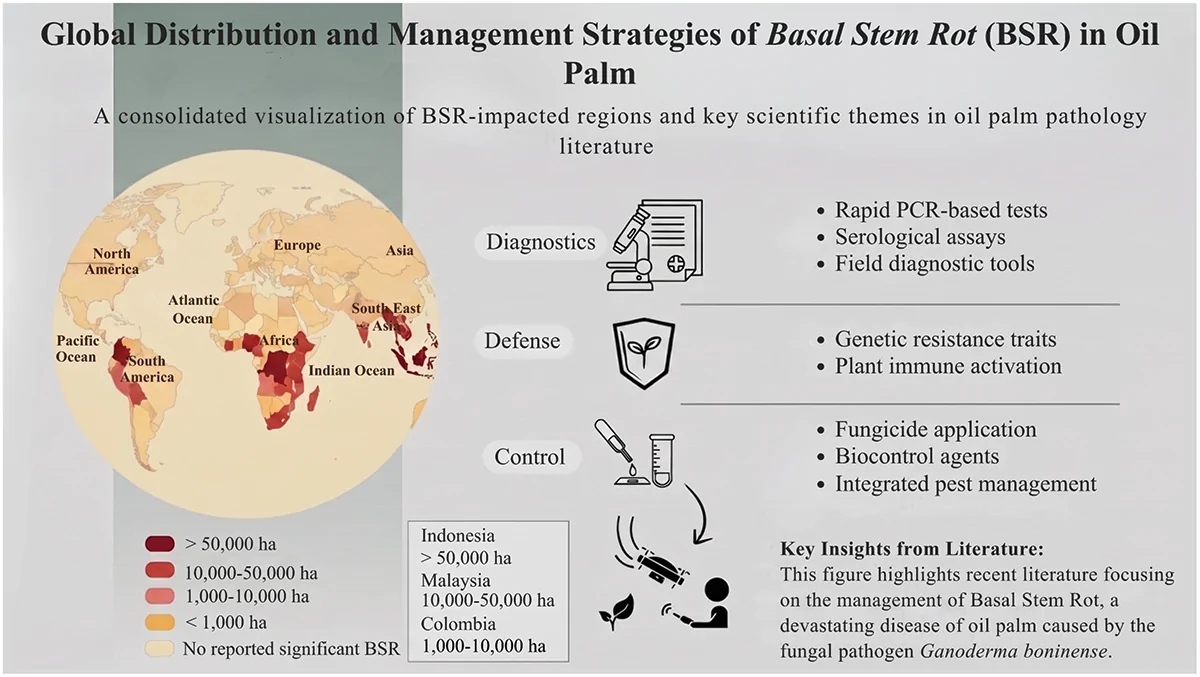
Global distribution of basal stem rot (BSR) caused by *Ganoderma boninense* and key scientific tools for its management. The map highlights regions with reported high, moderate, and low incidence based on literature synthesis^4,15,16^ and other regional reports.

This review compiles and critically evaluates a broad range of diagnostic tools from classical morphological identification to advanced molecular (qPCR, LAMP, CRISPR), spectroscopic, sensor-based, and remote sensing methods. A consolidated summary of 80 representative techniques, including their sensitivity, cost, field applicability, and limitations, is presented in Table 3. The review further discusses oil palm defense responses at the molecular and physiological levels (with emphasis on salicylic acid signaling and secondary metabolites), outlines the pathogen’s life cycle to identify vulnerable stages, and examines integrated disease management (IDM) strategies that combine agronomic, chemical, biological, and genetic approaches. By synthesizing the latest peer-reviewed findings, this section aims to bridge current practices with emerging needs for researchers and policymakers.

**Table 3.** Overview of 80 diagnostic techniques for *Ganoderma boninense* detection in oil palm.

Technique Name	Detection Target	Mechanism of Action	Sensitivity/Commercial Availability	Cost/Time to result	Field Applicability	Key Benefits/Portability	Major Limitations	Stage	Reference
Molecular Diagnostics									
Conventional PCR	DNA (ITS region)	Thermal cycling with specific primers	Moderate (100 pg)/Yes	Medium/2–3 hrs	Low	High specificity/Benchtop	Requires thermal cycler	Both	^17^
Nested PCR	DNA (ITS, β-tubulin)	Two-step PCR for enhanced sensitivity	High (10 pg)/Yes	Medium/4–5 hrs	Low	Greater sensitivity/Benchtop	Time-intensive	Asymptomatic	^1,18^
Quantitative PCR (qPCR)	DNA	Real-time fluorescence monitoring	High (1 pg)/Yes	Medium/1–2 hrs	Low	Quantitative, sensitive/Benchtop	Expensive equipment	Both	^1,19^
Loop-Mediated Amplification (LAMP)	DNA (ITS region)	Isothermal amplification	High (10 pg)/Yes	Low/30–60 min	Medium	Fast, portable/Handheld	Requires DNA extraction	Asymptomatic	^17,19^
Recombinase Polymerase Amplification (RPA)	DNA (ITS region)	Recombinase-mediated amplification	High (5 pg)/Limited	Medium/15–20 min	Medium	Rapid, field-adaptable/Handheld	Reagent stability issues	Asymptomatic	^1^
DNA Barcoding	DNA (ITS, LSU)	Sequencing of standardized markers	High (1 ng)/Yes	High/6–8 hrs	Low	Species identification/Benchtop	Bioinformatics required	Both	^1,17^
CRISPR-Cas12a	DNA (specific sequence)	Cas12a activation with fluorescence	Very High (<1 pg)/Limited	Medium/<1 hr	Medium–High	High specificity, field-ready/Handheld	Guide RNA design needed	Asymptomatic	^1^
DNA Microarrays	Multiple fungal genes	Hybridization on gene chips	High (100 pg)/Limited	High/8–12 hrs	Low	Comprehensive profiling/Benchtop	High cost, complex	Both	^1^
Droplet Digital PCR (ddPCR)	DNA	Droplet-based absolute quantification	Very High (1 copy/µL)/Yes	High/2–3 hrs	Low	Precise low-abundance detection/Benchtop	Costly (~USD 100/sample)	Asymptomatic	^17^
Multiplex PCR	Multiple loci	Simultaneous amplification of loci	High (10 pg)/Yes	Medium/2–3 hrs	Low	Multi-target detection/Benchtop	Primer interactions	Both	^1^
TaqMan Assays	DNA	Probe-based real-time detection	High (1 pg)/Yes	Medium/1–2 hrs	Low	High specificity/Benchtop	Probe optimization needed	Both	^17^
SYBR Green Assays	DNA	Non-specific dye-based detection	Moderate (50 copies)/Yes	Low/1–2 hrs	Low	Cost-effective/Benchtop	Non-specific binding	Both	^17,19^
High-Resolution Melting (HRM)	DNA	Post-PCR melting curve analysis	High (5 pg)/Limited	Medium/2–3hrs	Low	Sequence variation detection/Benchtop	Temperature control critical	Both	^17^
Digital PCR	DNA	Absolute quantification without standards	Very High (5 copies/µL)/Yes	High/2–3 hrs	Low	High precision/Benchtop	Expensive equipment	Asymptomatic	^1^
Whole-Genome Sequencing (WGS)	Entire genome	Full genomic sequencing	Very High (fM)/Limited	Very High/12-24hrs	Low	Strain differentiation/Benchtop	High cost (~USD 1,000)	Both	^1^
Biochemical Diagnostics Techniques									
Enzyme-Linked Immunosorbent Assay (ELISA)	Proteins/Antigens	Antibody-antigen binding	High (1 ng/mL)/Yes	Medium/2 hrs	Medium	High specificity/Benchtop	Cold chain needed	Both	^17,19^
Western Blot	Proteins	Protein separation and detection	High (10 ng)/Yes	Medium/6–8 hrs	Low	Confirmatory analysis/Benchtop	Labor-intensive	Both	^17^
Immunostrip Assays	Antigens	Lateral flow antibody detection	Moderate (5 ng/mL)/Yes	Low/15 min	High	Portable, rapid/Handheld	Lower sensitivity	Both	^1,17^
Proteomics (LC-MS/MS)	Fungal proteins	Mass spectrometry protein profiling	Very High (100 pg)/Limited	High/6–8 hrs	Low	Identifies PR proteins/Benchtop	High cost (~USD 500)	Both	^1^
2D Gel Electrophoresis	Proteins	Two-dimensional protein separation	High (1 µg)/Limited	Medium/8–10 hrs	Low	Detailed protein mapping/Benchtop	Skilled personnel required	Both	^19^
Ergosterol Quantification	Ergosterol	GC-MS based fungal biomass marker	High (0.1 µg/g)/Limited	High/4–6 hrs	Low	Fungal-specific/Benchtop	Complex prep	Both	^17^
VOC Metabolite Profiling	VOCs	GC-MS detection of fungal volatiles	High (0.01 ppm)/Limited	High/4–6 hrs	Low	Early detection/Benchtop	Costly instrumentation	Asymptomatic	^1^
Spectroscopy									
Near-Infrared Spectroscopy (NIRS)	Leaf chemistry	Near-IR light absorption	High (85%)/Yes	Medium/5–10 min	High	Non-destructive/Handheld	Calibration required	Both	^17,19^
Fourier Transform Infrared Spectroscopy (FTIR)	Fungal cell wall	IR spectrum of chemical bonds	High (80%)/Yes	Medium/5–10 min	Medium	Non-invasive/Benchtop	Database calibration	Both	^1,19^
Raman Spectroscopy	Root/leaf chemicals	Inelastic scattering of laser light	High (80%)/Limited	Medium/1 hr	Medium	Label-free/Benchtop	Weak signal in tissues	Both	^1^
Fluorescence Spectroscopy	Autofluorescent compound	Light emission detection	Moderate (75%)/Yes	Medium/5–10 min	Medium	Rapid/Benchtop	Dark conditions needed	Both	^1^
UV–Visible Spectroscopy	Pigments	Absorption in UV-Vis range	Moderate (70%)/Yes	Low/5–10 min	Medium	Cost-effective/Benchtop	Non-specific	Both	^1^
Hyperspectral Imaging	Reflectance	Multi-wavelength reflectance mapping	High (85%)/Limited	High/1 hr	Medium	Detailed biochemical data/Benchtop	High cost (~USD 30,000)	Both	^1,17^
Laser-Induced Breakdown Spectroscopy (LIBS)	Elemental composition	Laser-induced plasma emission	Moderate (70%)/Limited	Medium/1 hr	Medium	Elemental profiling/Benchtop	Limited sensitivity	Both	^1,19^
Terahertz Spectroscopy	Water content	Terahertz wave absorption	Moderate (65%)/No	High/1–2 hrs	Low	Non-invasive/Benchtop	Low resolution	Both	^1^
Photoacoustic Spectroscopy	Volatile compound	Sound wave generation by light	Moderate (70%)/Limited	Medium/1 hr	Medium	Sensitive to volatiles/Benchtop	Requires controlled settings	Both	^17,19^
Sensor-Based Diagnostics Techniques									
Surface Plasmon Resonance (SPR)	Antigens	Refractive index change detection	High (0.1 ng/mL)/Limited	High/10–15 min	Medium	Real-time/Benchtop	High cost (~USD 50,000)	Both	^1^
Quartz Crystal Microbalance (QCM)	Fungal biomass	Mass change on crystal surface	High (1 µg/cm^2^)/Limited	Medium/15–20 min	Medium	Sensitive/Benchtop	Surface prep required	Both	^19^
Amperometric Biosensors	Enzyme activity	Electrical current measurement	High (0.5 nA/µM)/Limited	Medium/10–15 min	Medium	Rapid/Benchtop	Enzyme stability issues	Both	^1^
Optical Biosensors	Metabolites	Light-based detection	High (85%)/Limited	Medium/10–15 min	Medium	High specificity/Benchtop	Light interference	Both	^17,19^
Bioimpedance Sensors	Tissue conductivity	Electrical impedance changes	High (5% change)/Limited	Medium/5–10 min	High	Real-time/Handheld	Environmental interference	Both	^1,17^
Capacitance Sensors	Dielectric shifts	Capacitance measurement	High (80%)/Limited	Medium/5–10 min	High	Non-invasive/Handheld	Moisture sensitivity	Both	^17^
Voltammetric Sensors	Redox reactions	Voltage changes due to reactions	High (0.1 µM)/Limited	Medium/10–15 min	Medium	Sensitive/Benchtop	Electrode fouling	Both	^19^
Electrochemical Impedance Spectroscopy (EIS)	Metabolic activity	Impedance spectrum analysis	Very High (90%)/Limited	Medium/20 min	Medium	Metabolic profiling/Benchtop	Environmental variability	Both	^1,17^
Field Studies Diagnostics Techniques									
Soil Metagenomics	Fungal DNA	Next-gen sequencing of soil DNA	Very High (99%)/Limited	High/24–48 hrs	Medium	Comprehensive profiling/Benchtop	High cost (~USD 200)	Both	^1^
16S rRNA Sequencing	Bacterial DNA	Sequencing 16S rRNA gene	High (95%)/Yes	High/24–48 hrs	Medium	Microbial insight/Benchtop	Indirect detection	Both	
Fungal ITS Sequencing	Fungal DNA (ITS)	Sequencing of ITS regions	Very High (98%)/Limited	High/24–48 hrs	Medium	Species-specific/Benchtop	Primer design critical	Both	^1,17^
Soil DNA Barcoding	Fungal DNA	Standardized marker sequencing	High (1 ng)/Limited	High/6–8 hrs	Medium	Species identification/Benchtop	Sampling inconsistency	Both	^17^
Phospholipid Fatty Acid (PLFA) Analysis	Fungal biomass	Fatty acid profiling	High (0.5 µg/g)/Limited	Medium/4–6 hrs	Medium	Rapid biomass estimate/Benchtop	Lacks specificity	Both	^17,19^
Microbial Community Profiling	Microbial DNA	Shotgun metagenomic sequencing	Very High (90%)/Limited	High/24–48 hrs	Medium	Holistic insight/Benchtop	High cost (~USD 300)	Both	^1^
UAV Phenotyping	Canopy changes	High-res camera imaging	High (85%)/Yes	High/1 hr	High	Scalable/Remote	Data processing intensive	Both	^19^
Drone Multispectral Imaging	Reflectance	Multispectral wavelength capture	High (88%)/Yes	High/1 hr	High	Detailed stress mapping/Remote	Cloud cover limitation	Both	^1,19^
Climate Modeling	Infection risk	Humidity/temp data integration	Moderate (75%)/Yes	Medium/1–2 d	High	Predictive capability/Remote	Model accuracy varies	Both	^1^
Disease Risk Mapping	Disease hotspots	Geospatial data integration	High (80%)/Yes	Medium/1–2 d	High	Regional monitoring/Remote	Needs frequent updates	Both	^17^
Thermal Imaging (UAV)	Temperature anomalies	Infrared thermal detection	High (82%)/Yes	High/1 hr	High	Non-invasive/Remote	Altitude resolution loss	Both	^17,19^
Satellite Phenotyping	Canopy health	MODIS-based reflectance	Moderate (70%)/Yes	High/2–3 d	Very High	Large-scale monitoring/Remote	Low temporal resolution	Both	^1^
Thermal Imaging	Temperature changes	Infrared detection of heat anomalies	High (82%)/Yes	Medium	High	Non-invasive/Handheld	Limited specificity	Both	^19^
RGB Camera Scoring	Frond yellowing	Color-based symptom assessment	Moderate (78%)/Yes	Low/5–10 min	High	Simple, rapid/Handheld	Non-specific symptoms	Symptomatic	^19^
Hyperspectral Imaging	Reflectance	Multi-wavelength reflectance mapping	High (85%)/Limited	High/1 hr	Medium	Detailed biochemical data/Benchtop	High cost (~USD 30,000)	Both	^17^
Machine Learning Classification	Image features	CNN-based image analysis	High (90%)/Limited	Medium/1–2 hrs	Medium	Automated, accurate/Benchtop	Large training datasets	Both	^1^
Automated Symptom Scoring	Visual cues	Algorithmic severity scoring	High (85%)/Limited	Medium/1–2 hrs	Medium	Consistent scoring/Benchtop	Data curation needed	Symptomatic	^17^
VOC-Based Diagnostics Techniques									
E-Nose (Metal Oxide)	VOCs (ethanol)	Conductivity change detection	High (92%)/Yes	Medium/10 min	High	Non-destructive/Handheld	Humidity interference	Asymptomatic	^17^
E-Nose (Conducting Polymer)	VOCs (aldehydes)	Resistance change detection	High (88%)/Yes	Medium/10 min	High	Rapid/Handheld	Humidity sensitivity	Asymptomatic	^17,19^
E-Nose (Quartz Microbalance)	VOCs	Mass change detection	High (90%)/Limited	Medium/15 min	Medium	High specificity/Benchtop	Frequent recalibration	Asymptomatic	^17^
E-Nose (Surface Acoustic Wave)	VOCs	Frequency shift detection	High (87%)/Limited	Medium/15 min	Medium	Sensitive/Benchtop	Temperature effects	Asymptomatic	^17^
Optical E-Nose	VOCs	Colorimetric array detection	High (85%)/Limited	Medium/10 min	Medium	Visual output/Benchtop	Less robust in field	Asymptomatic	^17,19^
CNN for E-Nose	VOC patterns	Neural network classification	Very High (95%)/Limited	Medium/15 min	Medium	High accuracy/Benchtop	Calibration needed	Asymptomatic	^1^
SVM for E-Nose	VOC data	Support vector classification	High (93%)/Limited	Medium/15 min	Medium	Noise robust/Benchtop	High computation	Asymptomatic	^19^
Random Forest for E-Nose	VOC data	Ensemble tree classification	High (91%)/Limited	Medium/15 min	Medium	Handles large data/Benchtop	Extensive training	Asymptomatic	^1^
PCA for E-Nose	VOC data	Dimensionality reduction	High (89%)/Limited	Low/10 min	Medium	Data simplification/Benchtop	Limited discrimination	Asymptomatic	^19^
Remote Sensing Diagnostics Techniques									
Sentinel-2 Imaging	Canopy stress	Multispectral imaging (10 m)	High (80%)/Yes	High/2–3 d	Very High	Large-scale monitoring/Remote	Cloud interference	Both	^1^
Landsat-8	Canopy health	Multispectral imaging (30 m)	Moderate (75%)/Yes	High/2–3 d	Very High	Broad coverage/Remote	Low resolution	Both	^17^
LiDAR	Structural changes	Laser-based height mapping	High (85%)/Limited	High/1–2 d	High	Detailed structural data/Remote	High cost	Both	^17^
MODIS	Canopy health	Coarse-scale reflectance	Moderate (70%)/Yes	High/2–3 d	Very High	Vast area monitoring/Remote	Low detail	Both	^17^
Hyperspectral Satellite Imaging	Biochemical stress	Multi-wavelength reflectance	High (82%)/Limited	High/2–3 d	Very High	Detailed stress mapping/Remote	Data processing intensive	Both	^19^
Spatial Mapping	Disease hotspots	GPS-based spatial data	High (80%)/Yes	Medium/1–2 d	High	Hotspot identification/Remote	Update frequency needed	Both	^1^
GIS-Based Disease Tracking	Disease spread	Geospatial data integration	High (85%)/Yes	Medium/1–2 d	High	Spread monitoring/Remote	Data integration complex	Both	
Geostatistical Analysis	Spatial patterns	Statistical modeling of spread	High (78%)/Yes	Medium/1–2 d	High	Predictive modeling/Remote	Regional data variability	Both	^17,19^
Genetic Diagnostics Techniques									
Metabarcoding	Fungal ITS regions	Targeted sequencing of ITS	Very High (99%)/Limited	High/24–48 hrs	Medium	Species-specific/Benchtop	Bioinformatics needed	Both	^17^
Amplicon Sequencing	Specific loci	PCR-based sequencing	High (1 pg)/Limited	High/24–48 hrs	Medium	Targeted resolution/Benchtop	Primer quality critical	Both	^1^
RNA Sequencing	Fungal RNA	Transcriptome profiling	High (95%)/Limited	High/24–48 hrs	Low	Gene expression insight/Benchtop	High cost (~USD 200)	Both	^19^
Single-Molecule Real-Time Sequencing (SMRT)	Fungal genome	Long-read sequencing	Very High (98%)/Limited	High/12–24 hrs	Low	Improved assembly/Benchtop	Costly sequencing depth	Both	^17^
Nanopore Sequencing	Fungal DNA	Portable real-time sequencing	High (97%)/Limited	Medium/2 hrs	High	Field-deployable/Handheld	5–10% error rate	Both	^17^
Shotgun Metagenomics	All DNA	Unbiased sequencing of all DNA	Very High (96%)/Limited	High/24–48 hrs	Medium	Holistic profiling/Benchtop	High cost (~USD 500)	Both	^19^
Droplet Digital PCR (ddPCR)	DNA	Droplet-based quantification	Very High (1 copy/µL)/Yes	High/2–3 hrs	Low	Precise low abundance/Benchtop	Costly (~USD 100)	Asymptomatic	^17^
Multiplex PCR	Multiple loci	Simultaneous locus amplification	High (10 pg)/Yes	Medium/2–3 hrs	Low	Multi-target/Benchtop	Primer interactions	Both	^17,19^
TaqMan Assays	DNA	Probe-based real-time detection	High (1 pg)/Yes	Medium/1–2 hrs	Low	High specificity/Benchtop	Probe optimization	Both	^17^
SYBR Green Assays	DNA	Dye-based real-time detection	Moderate (50 copies)/Yes	Low/1–2 hrs	Low	Cost-effective/Benchtop	Non-specific binding	Both	^17^
High-Resolution Melting (HRM)	DNA	Melting curve analysis	High (5 pg)/Limited	Medium/2–3 hrs	Low	Sequence variation/Benchtop	Temperature control	Both	^1,19^
Digital PCR	DNA	Absolute quantification	Very High (5 copies/µL)/Yes	High/2–3 hrs	Low	High precision/Benchtop	Expensive equipment	Asymptomatic	^17,19^
Whole-Genome Sequencing (WGS)	Entire genome	Full genomic sequencing	Very High (fM)/Limited	Very High/12–24 hrs	Low	Strain differentiation/Benchtop	High cost (~USD 1,000)	Both	^17,19^

## 3. Biology, Ecology and Epidemiology of *G. boninense*

### 3.1. Taxonomy and Phylogenetic Context

*G. boninense* belongs to the Kingdom Fungi, Phylum Basidiomycota, and Order Polyporales, a group renowned for wood-decaying capabilities.^20^ Within the genus Ganoderma, it is closely related to *G. lucidum* and *G. zonatum*, but differs in host specificity and aggressiveness toward oil palm.^14^ Phylogenetic analyses based on internal transcribed spacer (ITS) regions reveal significant divergence that influences host preference, with *G. boninense* primarily targeting *Elaeis guineensis*.^21,22^ This divergence underscores the importance of species-specific diagnostics and control strategies, as pathogenicity traits vary across the genus.

### 3.2. Life Cycle of *G. boninense*

Each stage of *G. boninense*’s life cycle plays a critical role which defines its survival prospect in oil palm ecosystems. Spore production and germination is the first stage of infection is the development of fruiting bodies named basidiocarps at the stem base of infected or dying oil palms. Basidiocarps give rise to basidiospores that either float in the air or settle into the soil, where they do well under high humid conditions with adequate organic matter.^23^ Mycelial growth and rhizomorph formation comes after germination, basidiospores grow into primary mycelium and start to cover the host root. The hyphae eventually combine to form rhizomorphs, which make cable like thick structure enabling swift dispersion of pathogen throughout the oil palm’s roots.^12^ Root invasion and host colonization is the mycelium passes through wounds or natural cracks in the roots before moving into the vascular system, causing the stem to rot at its base. Palm colonization can go unnoticed for many months or years, while cell-destroying enzymes and toxins work inside the host tissue. Fruiting body formation is the appearance of basidiocarps on dead or dying trees lets farmers know about the severity of infection and incidence of disease symptoms. Spore release and dispersal is spores spread into the environment with the help of wind or water, allowing the disease to start over in other healthy palms.^24^

Figure 2 shows a detailed illustration of the two ecological roles of *G. boninense* and its infection route and explains how it manages to persist in oil palm ecosystems. It compares the pathogenic effects of the fungus, with its saprophytic role in nutrient cycling in tropical soils. Asexual reproduction via chlamydospores or mycelial fragments also contributes to persistence in soil and debris. The diagram identifies the steps of the infection process, starting with the entry of the roots to the degradation of the tissues, and marks the important stages, such as the growth of mycelium and the generation of toxins. Combining ecological and pathological knowledge, Figure 2 can be utilized as an essential resource by scientists who plan to break the disease cycle. The biological, ecological, and epidemiological characteristics of *G. boninense* as presented in Table 1, describe the mechanisms of germination of basidiospores to latent infection with diagnostic and control implications. This list emphasizes the flexibility of the fungus to live in high-moisture conditions as well as its long-term saprophytic existence, making it difficult to manage. Table 1 can steer interventions based on the mapping of traits, detection methods (e.g., qPCR) and control tactics (e.g., soil drenching). It deals with the billion-dollar per year economic impact of *G. boninense* by revealing the existence of its vital lifecycle vulnerabilities.^41,42^

**Figure 2.**
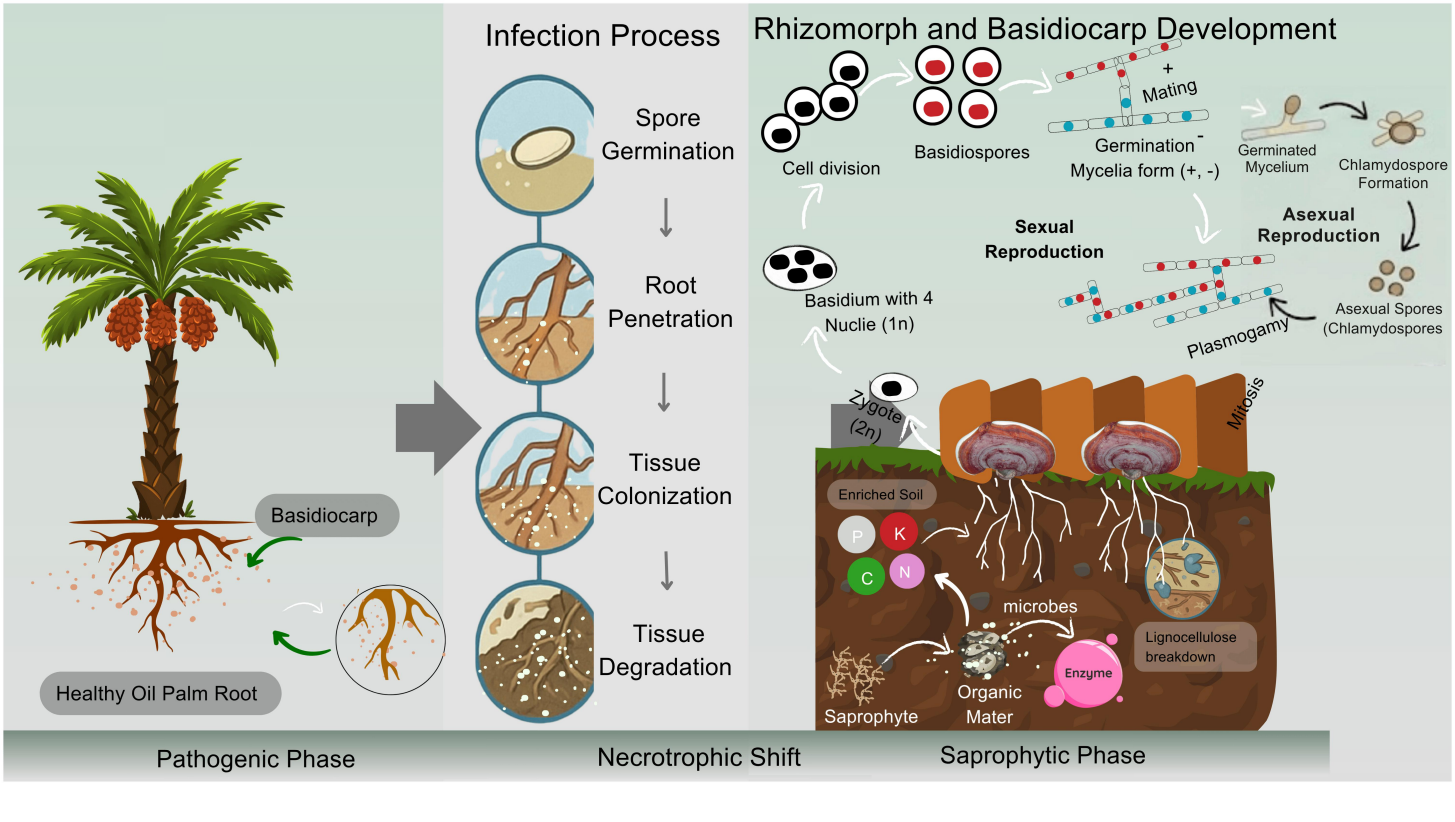
Life cycle phases of *Ganoderma boninense* in oil palm, illustrating both pathogenic (root infection to basidiocarp formation) and saprophytic roles. Note the basidiocarp (fruiting body typical of polypore fungi) and include asexual structures where relevant (e.g., chlamydospores or mycelial fragments).

**Table 1.** Key biological, ecological, and epidemiological traits of *Ganoderma boninense* in BSR.

Trait	Mechanism	Diagnostic Relevance	Control Implications	Limitations	Reference
Spore Germination	Basidiospores germinate under high humidity (>80%) and temperatures of 25–30°C, initiating primary mycelium formation in soil or on wounded roots.	Detection of spores in soil or air samples via spore traps or qPCR indicates early infection risk.	Informs timing of preventive measures, such as fungicide applications or soil moisture management.	Airborne spore presence does not always correlate with infection; it requires integration with soil and host data.	^12^
Primary Mycelial Growth	Germinated spores produce thread-like hyphae that colonize root surfaces, forming a network for nutrient acquisition and host invasion.	Microscopic examination or ITS sequencing of mycelia in root samples confirms early fungal presence.	Early detection allows for targeted root zone treatments, such as bio fungicides.	Sampling small root fragments is labor-intensive and may miss early infections due to uneven distribution.	^1^
Rhizomorph Formation	Hyphae aggregate into thick, rope-like rhizomorphs, enabling systemic spread between roots over distances up to 2 meters.	Presence of rhizomorphs in soil or on roots, detectable via soil excavation, signals active spread.	Rhizomorph-targeted control, such as soil drenching with fungicides, can limit spread.	Rhizomorphs are often subsurface, making detection challenging without invasive sampling techniques.	^25,26^
Root Penetration Mechanisms	Entry occurs through wounds or natural root cracks, facilitated by mechanical force and enzymatic softening of cell walls.	Root tissue analysis for fungal DNA or enzyme activity (e.g., cellulases) indicates penetration sites.	Protective root barriers or wound treatments (e.g., fungicide pastes) can reduce entry points.	Asymptomatic entry complicates early diagnosis; wounds are often micro-scale and difficult to monitor.	^12,27^
Enzymatic Degradation	Production of cellulases, lignin peroxidases, and laccases degrades lignocellulosic tissues, enabling vascular invasion and tissue decay.	Elevated enzyme levels in infected tissues, detectable via biochemical assays, confirm active degradation.	Enzyme inhibitors or resistant cultivars with fortified cell walls can mitigate decay.	Enzyme activity varies with infection stage, leading to inconsistent diagnostic results across samples.	^28,29^
Toxin Production	Ganoderic acids and other secondary metabolites induce host cell necrosis and suppress immune responses.	Detection of ganoderic acids via HPLC in infected tissues serves as a biomarker for active infection.	Toxin-neutralizing agents or resistant varieties can reduce necrosis.	Limited understanding of ganoderic acid biosynthesis pathways hinders targeted control strategies.	^30,31^
Basidiocarp Development	Fruiting bodies form on the stem base of dead or dying trees, indicating advanced systemic colonization.	Visible basidiocarps are a late-stage diagnostic marker, identifiable through visual inspection.	Removal of infected trees with basidiocarps prevents further spore release.	Basidiocarps appear late, limiting their utility for early detection and proactive control measures.	^32,33^
Host Vascular Colonization	Mycelia spread through vascular tissues, causing xylem blockage and basal stem rot over months to years.	Vascular tissue sampling and PCR-based detection confirm fungal presence in xylem.	Systemic fungicides or biocontrol agents targeting vascular spread can slow progression.	Sampling vascular tissues is invasive and impractical for large-scale monitoring in plantations.	^33^
Latent Infection Periods	Asymptomatic colonization can persist for 1–2 y before visible symptoms emerge, driven by slow mycelial growth.	Molecular diagnostics (e.g., qRT-PCR) in asymptomatic palms detect latent infections.	Early intervention during latency, such as biocontrol, can prevent symptom onset.	Latency duration varies with environmental conditions, complicating predictive models for disease onset.	^12,34^
Saprophytic Survival	Survives saprophytically in dead plant tissues and soil organic matter, persisting between planting cycles.	Soil metagenomics identifies *G. boninense* in organic residues, indicating inoculum reservoirs.	Removal of dead wood and organic debris reduces saprophytic reservoirs.	Complete removal of organic matter is labor-intensive and often impractical in large plantations.	^12,14^
Soil Persistence of Inoculum	Basidiospores and chlamydospores remain viable in soil for years, serving as long-term inoculum sources.	Soil DNA extraction and sequencing detect persistent inoculum, guiding risk assessment.	Soil fumigation or biocontrol agents can reduce inoculum levels.	Soil persistence varies with soil type and depth, making eradication inconsistent and challenging.	^12,27^
Airborne Dispersal of Spores	Basidiospores disperse via air currents, though limited in range, contributing to new infections.	Spore traps and air sampling detect airborne spores, indicating dispersal risk.	Windbreaks or spore-trapping barriers can limit aerial spread.	Quantifying long-distance dispersal is difficult, limiting predictive accuracy of spread models.	^27,35^
Waterborne Spread	Spores move with rainwater or irrigation, facilitating spread within and between fields.	Water sampling for spore DNA via PCR identifies waterborne transmission routes.	Improved drainage and irrigation management reduce waterborne spread.	Water movement patterns are unpredictable, complicating targeted control of waterborne transmission.	^12,27^
Spread via Contaminated Tools	Human-mediated spread occurs via contaminated tools, footwear, and machinery during pruning or harvesting.	Swabbing tools for fungal DNA confirms contamination risk.	Tool sterilization and hygiene protocols prevent human-mediated spread.	Compliance with hygiene protocols is inconsistent in large plantations, reducing effectiveness.	^27,36^
Environmental Triggers	High moisture (>80%) and temperatures (27–30°C) enhance spore germination, mycelial growth, and virulence.	Monitoring environmental conditions predicts infection risk, guiding diagnostic timing.	Climate-smart practices, such as drainage systems, mitigate environmental triggers.	Environmental variability across regions limits the universality of predictive models.	^12,27^
Agronomic Drivers	Dense planting and root proximity in monocultures facilitate root-to-root spread via rhizomorphs.	Mapping planting density identifies high-risk zones for targeted diagnostics.	Adjusting planting density and intercropping reduces spread.	Changing agronomic practices is costly and may conflict with yield optimization goals.	^7,12^
Asymptomatic Disease Progression	Disease progresses silently for years, with symptoms (e.g., frond yellowing) appearing late.	Advanced diagnostics (e.g., e-nose, LAMP) detect asymptomatic infections.	Early control during asymptomatic phase prevents severe damage.	Asymptomatic detection requires expensive tools, limiting scalability in resource-constrained regions.	^12,19^
Host Range and Specificity	Primarily infects oil palm, with limited reports on coconut and rubber, driven by host-specific enzymes.	Host range informs diagnostic specificity, focusing on oil palm.	Crop rotation with non-hosts reduces infection pressure.	Limited host range data restricts understanding of cross-species transmission risks.	^27,37^
Resistance Breakdown	No fully resistant palm varieties exist; partial resistance breaks down under high inoculum pressure.	Resistance screening via phenotyping identifies partial resistance for breeding.	Breeding programs focus on partial resistance, but progress is slow.	Genetic resistance is temporary, as *G. boninense* adapts to overcoming host defenses over time.	^38,39^
Host Immune Evasion	Effectors and detoxifying enzymes suppress host oxidative burst and immune responses.	Proteomic analysis of infected tissues identifies effectors as diagnostic targets.	Immune-boosting treatments (e.g., salicylic acid) enhance host defense.	Effector mechanisms are poorly characterized, limiting development of immune-based control strategies.	^40^

### 3.3. Pathogenic Mechanisms

*G. boninense* uses several strategies to attack and harm its host. Entry and invasion, in most cases, the fungus infects by penetrating cells using both physical strength and enzymes that loosen walls near wounds or through root contact.^14^ Enzymatic arsenal is the pathogen releases cellulases, lignin peroxidases, and laccases that together break down wooden tissues and aid in the infection’s spread throughout the plant, using the vascular system.^43^ For toxin production the fungus uses ganoderic acids, along with other secondary metabolites, to trigger cell death in the host and hamper the immune response, accelerating the disintegration of tissues.^44^ In evasion of host defenses, *G. boninense* uses detoxification enzymes and substances that prevent generation of reactive oxygen species which allows the fungus to live longer in the host tissues.^14^

### 3.4. Epidemiological Patterns of BSR

Multiple routes of transmission facilitate the spread of BSR in particular patterns. Primary infection sources are planting infected materials, exposing the field to contaminated soil or debris, and conducting weeding or pruning without precautions can cause BSR outbreaks (Bar-Joseph et al.^45^ Modes of spread is the transmission of BSR largely happens through rhizomorphs and mycelial growth. BSR may be transported by rain or irrigation water while air drift rarely contributes to BSR transmission. People can easily spread the disease, mostly by using the same tools, stepping on infected areas, or moving with harvesters by Singh and Kumar.^46^ Disease progression in the field is the first signs of BSR are usually found in clustered areas, where these spots grow outwards over the years. Figure 2 shows the ecology of *G. boninense* as a pathogen and saprophyte, as well as the process of its infection in oil palm. It shows the pathogenic cycle of the fungus, basidiospores germination till the invasion of roots and tissues degradation, which is maintained by enzymatic and toxin mechanisms. At the same time, it emphasizes the saprophytic breakdown of organic debris, which leads to nutrient cycling in the soil. The figure illustrates some critical phases, rhizomorph and basidiocarp development, that are important in the disease progression.

Many factors can change the results of BSR’s epidemiology. Older palms that reach 5 y or more are at higher risk of infection because larger roots and stress make them more susceptible.^39^ The risk of infection is highest during the rainy season because the wet weather increases moisture in the soil. Therefore, adjusting agricultural methods can help prevent further spread of BSR. Growing palms with adjoining legumes could lessen root contact and disease incidence, but research on this topic is still lacking. Primary spread occurs via root-to-root contact through rhizomorphs and infected plant debris/stumps. Basidiospores contribute to long-distance dispersal but their role in direct infection is considered secondary and environment-dependent.

### 3.5. Ecological Roles of *G. boninense*

Apart from its harmful role, *G. boninense* performs many important functions in nature. In saprophytic behavior, *G. boninense* feeds on dead plant remains and soil organic substances, supporting the decomposition of woody biomass and continuing its presence in fallow farms.^1^ Nutrient cycling is the Lignocellulosic breakdown by *G. boninense* circulates more carbon and nitrogen in tropical soils, helping to improve both soil structure and nutrient availability (Zhao and Riaz^47^). Mycorrhizal traits are Mycorrhiza-like interactions that take place among some *Ganoderma* species; however, *G. boninense*’s involvement is uncertain, as various studies suggest nutrient exchanges are conditional.^14^

### 3.6. Comparative Pathology with Fusarium and Phytophthora

*G. boninense* shares some pathogenic features with other soil-borne organisms such as Fusarium spp. (true fungi) and *Phytophthora* spp. (Oomycetes, fungus-like but not true fungi). All three penetrate roots and produce cell-wall-degrading enzymes. However, unlike *Phytophthora*, *G. boninense* primarily causes white-rot decay through extensive lignin and cellulose degradation rather than aggressive xylem invasion.^14^ Their target hosts also vary, as *G. boninense* mostly damage oil palm, *Fusarium* attacks cereals, and *Phytophthora* can infect various crops and ornamental plants.^14^ All three fungi cause oxidative stress in host plants and block the xylem tissues. *G. boninense* forms benzoic and salicylic acid, *Fusarium* forms fusaric acid, and *Ganoderma* forms ganoderic acids. All of them weaken the host’s defense system.^48^

### 3.7. Genomic and Molecular Insights

Genome sequencing uncovers the pathogenic nature of *G. boninense*. The genome contains a lot of carbohydrate-active enzymes (CAZymes), such as cellulases and ligninases, which help in breaking down plant cell walls.^49^ Ganoderic acids are produced by secondary metabolite clusters, and secreted effectors weaken the host’s defense system, making infection more efficient.^50^ Such genomic characteristics indicate *G. boninense* unique ability to cause disease, pointing out useful targets for its management.

Figure 3 shows that *G. boninense* life cycle can be started with the germination of the spores, which is induced by the high moisture levels common in tropical areas such as Southeast Asia. Fungus invasion causes growth suppression of the plants, whose roots get infected through the mechanical damage caused which introduces wounds that attract fungus infection to the roots and aggravated by soil compaction. The cycle is perpetuated through steady, hot, humidity that enhances sporocarp construction and fruiting and through monoculture planting which raises the risks of the disease. This is a cross-referencing process which ceaselessly emphasizes any environmental factors that stimulate the fan out of BSR.

**Figure 3.**
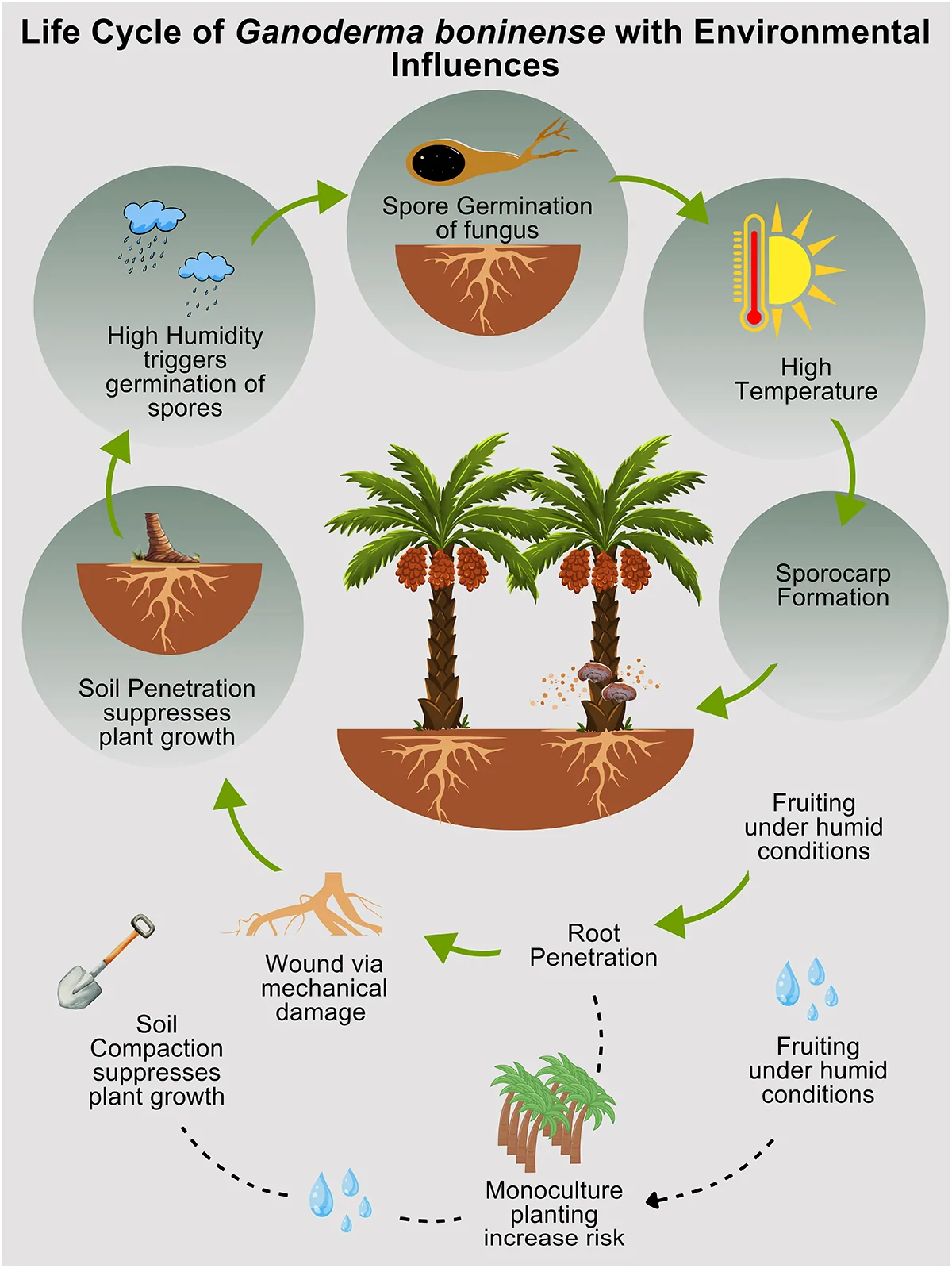
Life cycle of *G. boninense* and its environmental influences.

## 4. Host Defense Responses to BSR

*G. boninense* which causes BSR in oil palm has been creating billions of dollars in financial losses per year. Since the disease starts without symptoms and later on leads to quick rotting, it is important to study host–pathogen interactions to adopt pertinent approaches for its management.^4^ Understanding the molecular and physiological defense mechanisms of oil palm against *G. boninense* is important for developing environmentally friendly approaches to manage the biotic stress of oil palm diseases.^14^ While some mechanisms are well-documented in oil palm, others are inferred from related pathosystems and require species-specific validation.

### 4.1. Defense Signaling Pathways

Oil palm uses a network of complex signaling pathways to fight infection by *G. boninense*. Salicylic Acid (SA) Pathway: In response to biotrophic pathogen infection, SA helps in the rapid detection of *G. boninense* leading to develop systemic acquired resistance (SAR) and up-regulation of pathogenesis-related (PR) genes, e.g., EgPR1upregulation documented in oil palm.^14,51^ Transcriptomic studies of infected oil palm showed high SA levels within a day of *G. boninense* infection that corresponds to the activation of defense-related genes. Jasmonic Acid (JA) and ethylene (ET) Pathways: JA and ET pathways are mainly involved in protecting plants against the necrotrophic phase and coordinate through the expression of defense. ET reduces oxidative stress by strengthening cell walls with lignin in late stages of infection. Crosstalk and Fine-Tuning: At the beginning of infection, SA and JA/ET work against each other, while as necrosis advances, they begin to work together. Studies looking at hormone levels have revealed that SA take the lead during the early biotrophic phase, whereas JA and ET dominate during necrotrophic phase showing that oil palm defense system is well adopted to hormonal reconfiguration under biotic stress.^52,53^ Figure 4 describes signaling pathways of salicylic acid-mediated SAR and the jasmonic acid-mediated necrotrophic defenses. It has mapped signaling cascades of salicylic acid and jasmonic acid besides profiling metabolite production and gene expression in infected oil palm. This illustration shows the dynamic interactions of defense responses, from early recognition of the pathogen to late-stage necrotrophic resistance.

**Figure 4.**
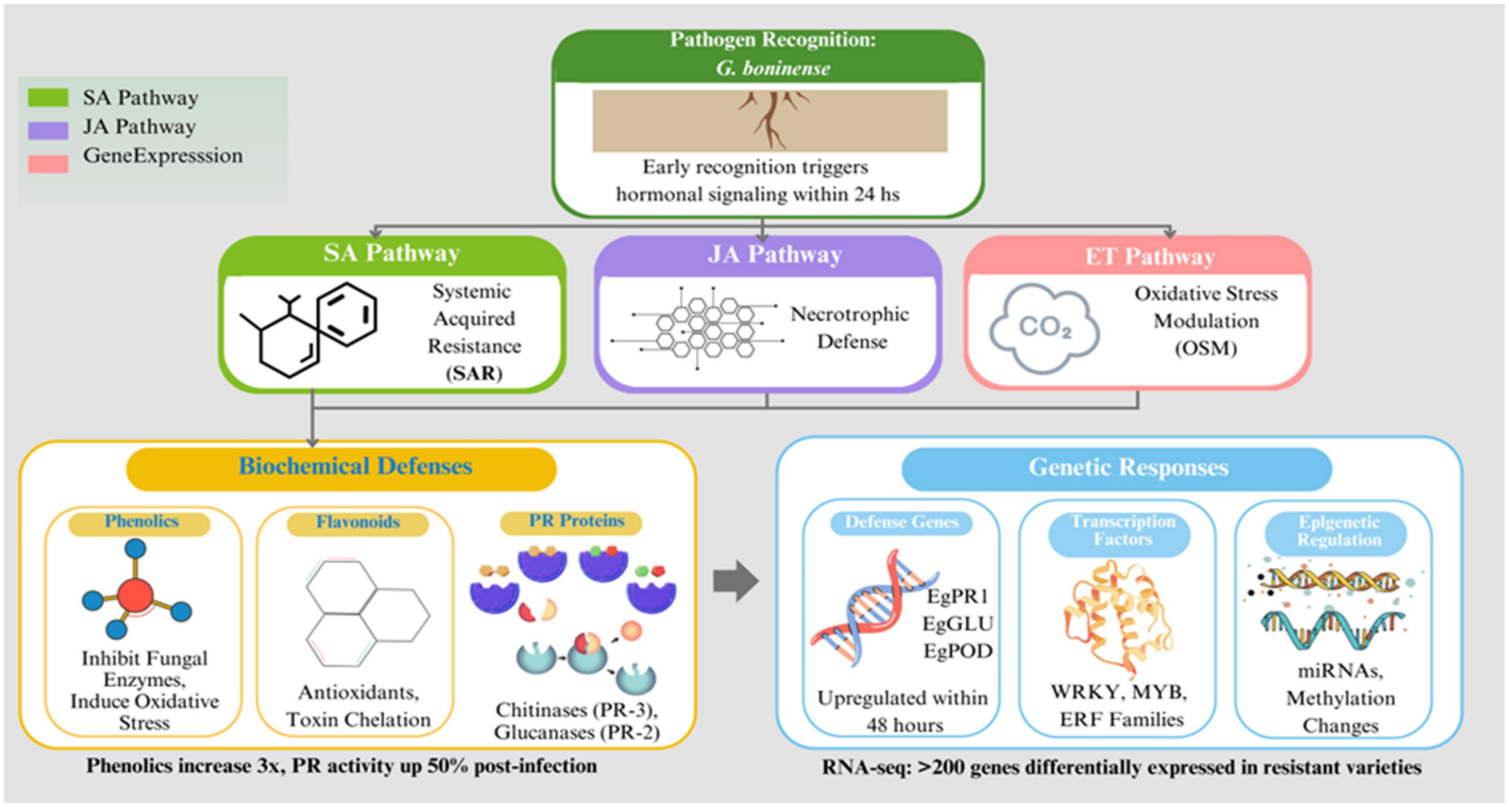
Signaling pathways and biochemical defense responses of oil palm against *G. boninense* infection, highlighting salicylic acid-mediated systemic acquired resistance (SAR) and jasmonic acid-mediated responses. Adapted and synthesized from key oil palm transcriptomic and hormonal studies.^54^

### 4.2. Biochemical Defenses and Metabolite Accumulation

Oil palm uses secondary metabolites and PR proteins to accumulate and defend against *G. boninense* infection. Phenolic Compounds: They work as obstacles to fungal enzymes and cause higher oxidative stress and their levels rise threefold in oil palm after *G. boninense* infection. Flavonoids: As antioxidants, flavonoids bind to fungal toxins and hold back hyphal growth, with the highest levels being found in vascular tissues during the early phase of infection.^55^ Pathogenesis-Related Proteins: In oil palm, the infection triggers a rise in both chitinase (PR-3) and β-1,3-glucanase enzymes. Defensins and oxidases are involved in fortifying defense by tightly connecting the cell wall and disabling fungal toxins.^56^ Moreover, upregulation of PR protein activity (up to 50% higher) has been reported within 1 week of infection in oil palm, These responses have been observed in oil palm root tissues following *G. boninense* challenge.^54^

Figure 4 demonstrates how biochemical responses (phenolic and flavonoid accumulation) counter fungal toxins in infected oil palm and clarifies the molecular and biochemical pathways that are important in the development of resistance against BSR. Table 2 summarizes hormonal signaling, oxidative bursts, as well as the accumulation of secondary metabolites, along with the key molecules such as salicylic acid and EgPR1.

**Table 2.** Host defense responses of oil palm to *G. boninense.*

Defense Type	Mechanism	Key Molecules/Genes	Effectiveness	Research Gaps	Reference
Hormonal Signaling (SA)	Activates salicylic acid pathway for early pathogen recognition and systemic acquired resistance (SAR).	*EgPR1*, *EgNPR1* (upregulated within 24 hr).	Moderate: Effective in early biotrophic phase.	Limited understanding of downstream SA signaling cascades and their role in necrotrophic stages.	^57^
Hormonal Signaling (JA)	Jasmonic acid pathway induces defense against necrotrophic phase, enhancing cell wall fortification.	*EgPR3* (chitinase), *EgPR4* (glucanase).	High: Strong response in necrotrophic phase.	Mechanisms of JA biosynthesis regulation in oil palm remain underexplored.	^58^
Hormonal Signaling (ET)	Ethylene pathway modulates oxidative stress and reinforces cell walls with lignin deposition.	*EgERF1*, ethylene-responsive factors.	Moderate: Enhances ROS management.	Specific ET-mediated pathways and their temporal dynamics need further investigation.	^59^
Hormonal Crosstalk	Antagonistic and synergistic interactions between SA and JA/ET pathways fine-tune defense responses.	*EgNPR1*, *EgERF1*.	Moderate: Balances biotrophic-necrotrophic responses.	Lack of detailed studies on cross-talk dynamics across infection stages.	^60^
Oxidative Burst	Production of reactive oxygen species (ROS) induces hypersensitive response to limiting fungal spread.	Superoxide dismutase, *EgCAT* (catalase).	High: Effective in early containment.	ROS regulation mechanisms and their impact on fungal suppression require further validation.	^54^
Reactive Nitrogen Species	Nitric oxide (NO) signaling enhances defense by promoting programmed cell death and enzyme activity.	*EgNOS* (nitric oxide synthase-like).	Moderate: Supports localized defense.	Role of NO in systemic resistance and its interaction with ROS signaling is poorly characterized.	^54^
Biochemical (Phenolics)	Accumulation of phenolic compounds in root tissues inhibits fungal enzymes and induces oxidative stress.	Phenolics, phenylpropanoid enzymes.	High: Forms infection barriers.	Quantitative profiling of phenolics across infection stages is lacking.	^12,27^
Biochemical (Flavonoids)	Flavonoids act as antioxidants, chelate fungal toxins, and suppress hyphal growth in infected tissues.	Flavonoids, chalcone synthase.	High: Reduces fungal growth.	Specific flavonoid pathways and their antifungal mechanisms need further elucidation.	^1,12^
PR Proteins (Chitinases)	Chitinases degrade fungal cell walls by hydrolyzing chitin, limiting mycelial growth.	*EgCHIT* (PR-3), upregulated post-infection.	High: Strong antifungal activity.	Field-level expression profiles of chitinases remain unvalidated.	^14,61^
PR Proteins (Glucanases)	β-1,3-glucanases degrade glucans in fungal cell walls, enhancing pathogen suppression.	*EgGLU* (PR-2), 50% activity increase.	High: Effective against fungal structures.	Lack of data on glucanase specificity and efficacy under varying environmental conditions.	^12,14^
PR Proteins (Peroxidases)	Peroxidases cross-link cell wall components, enhancing structural defense and ROS production.	*EgPOD* (peroxidase), upregulated.	Moderate: Strengthens cell walls.	Specific roles of peroxidase isoforms in BSR defense are not fully characterized.	^1,39^
PR Proteins (PPO)	Polyphenol oxidases oxidize phenolics to quinones, which are toxic to fungal pathogens.	Polyphenol oxidase enzymes.	Moderate: Contributes to toxicity.	Limited studies on PPO regulation and efficacy in field conditions.	^62^
Antifungal Defensins	Defensins disrupt fungal membrane integrity, inhibiting hyphal growth and spore germination.	*EgDEF1* (defensin-like protein).	Moderate: Localized antifungal effect.	Functional characterization of defensins in oil palm is incomplete.	^63^
Structural (Lignin)	Lignin deposition in root cortex reinforces cell walls, limiting fungal penetration.	Lignin, *EgPAL* (phenylalanine ammonia-lyase).	Moderate: Physical barrier formation.	In situ imaging data of lignin deposition in infected roots are lacking.	^64^
Structural (Callose)	Callose deposition at infection sites restricts fungal spread by sealing plasmodesmata.	*EgCalS* (callose synthase-like).	Moderate: Limits early spread.	Callose deposition dynamics and its role in systemic resistance need further study.	^65^
Transcriptional Regulation	WRKY and MYB transcription factors induce defense gene expression, orchestrating immune responses.	*EgWRKY33*, *EgMYB98*.	Moderate: Enhances defense gene activation.	Functional characterization of transcription factors is missing.	^66^
Transcriptional Regulation	Ethylene-responsive factors (ERFs) regulate PR gene expression during necrotrophic defense.	*EgERF1*, *EgERF2*.	Moderate: Supports necrotrophic response.	Specific ERF targets and their regulation mechanisms are underexplored.	^60^
Epigenetic Regulation	Post-transcriptional regulation via microRNAs silences or enhances defense gene expression.	miR482, miR398.	Unknown: Emerging field.	Role in defense is speculative; requires experimental validation.	^67^
Gene Co-expression Networks	RNA-seq reveals co-expressed defense gene clusters, indicating coordinated immune responses.	*EgPR1*, *EgGLU*, *EgPOD* clusters.	Moderate: Identifies resistance markers.	Limited integration with proteomic data to confirm co-expression functionality.	^40^
Membrane Receptor Kinases	Receptor-like kinases (RLKs) recognize pathogen-associated molecular patterns (PAMPs).	*EgNBS-LRR*, *EgRLK1*.	Moderate: Triggers early immunity.	Specific RLK-PAMP interactions in oil palm remain uncharacterized.	^68^
Sugar Metabolism Changes	Altered sugar metabolism diverts resources to defense, limiting fungal nutrient access.	*EgSPS* (sucrose phosphate synthase).	Low: Indirect defense mechanism.	Impact on fungal growth suppression and metabolic trade-offs is unclear.	^69^
Heat Shock Proteins	Heat shock proteins stabilize cellular processes under pathogen-induced stress.	*EgHSP70*, *EgHSP90*.	Low: Supports cellular homeostasis.	Specific roles in BSR defense and their regulation are poorly studied.	^70^
Endophyte-Triggered Immunity	Beneficial endophytes induce defense pathways, enhancing resistance to *G. boninense*.	*EgMYB98*, induced by *Trichoderma*.	Moderate: Enhances systemic resistance.	Mechanisms of endophyte-pathogen interactions in oil palm are underexplored.	^71^
Genotype-Dependent Response	Resistance varies across oil palm genotypes, with some showing higher PR gene expression.	*EgPR1* (higher in resistant varieties).	Moderate: Basis for breeding programs.	Limited genomic data on resistant genotypes hinders breeding efforts.	^38^
Crosstalk with Mycorrhiza	Mycorrhizal fungi may enhance defense by regulating stress-response genes.	*EgMT2* (metallothionein), *EgPOD*.	Unknown: Potential beneficial role.	Evidence of mycorrhizal benefits in BSR resistance is controversial and requires validation.	^38^

### 4.3. Genetic and Transcriptomic Responses

The interaction between oil palm and *G. boninense* causes complex gene expression patterns and regulatory networks. Early-Response Genes: As soon as infection occurs, genes like EgPR1, EgCHIT, EgGLU, and EgPOD are strongly activated as detected by RNA sequencing and this is apparent within 48 hr of infection. RNA-seq studies in oil palm have confirmed differential expression of over 200 genes in resistant versus susceptible varieties.^51,72^ Transcription factors belonging to WRKY proteins, myeloblastosis (MYB), and ethylene help organize these responses, boosting defense by switching on further defense mechanisms.^51,73^ Defense Gene Clusters and QTLs: Genomic mapping has revealed expression of QTLs which are associated with partial resistance during infection. Differential expression of more than 200 genes has also been reported using RNA-seq in resistant and susceptible varieties (Rathod et al.^74^). Epigenetic and Post-transcriptional Regulation: DNA methylation may help the plant recognize pathogens, and miRNAs could control the expression of particular defense genes. Though both processes are not very well explored, this genetic information provides breeders new targets to work with. Genetic regulation of resistance is emphasized by gene expression profile, such as EgPR1 and WRKY transcription factors in Fig. 4. The flow diagram brings together molecular and physiological information to uncover the dynamics of defense. This figure helps define the objective of increasing the resilience of oil palm to BSR.

### 4.4. Comparative Insights: Oil Palm vs. Other Crops

Comparative analysis with other host-pathogen systems provides valuable insights into oil palm defenses. Banana vs. *Fusarium oxysporum*: Both oil palm and banana exhibit vascular invasion by soil-borne fungi, with phenolic accumulation and JA/ET signaling as shared defense strategies.^75^ Transcriptomic studies in banana reveal similar upregulation of chitinases, mirroring oil palm responses.^76^ Grapevine vs. *Botrytis cinerea* or Rice vs. *Magnaporthe oryzae*: These systems highlight polygenic resistance and PR protein induction, with rice showing enhanced glucanase activity against blast disease, akin to oil palm’s response to *G. boninense*.^77^ Translational Insights: Gene editing tools like CRISPR and RNA interference (RNAi), successfully applied in rice for blast resistance, could be adapted for oil palm to enhance *EgPR1* expression, though species-specific optimization is required.^78,79^

## 5. Diagnostic Innovations for BSR Detection

This section provides an overview of diagnostic innovations for *G. boninense* detection. A comparative summary of key techniques, including sensitivity, cost, field suitability, and practicality for smallholders, is presented in Table 3. The following text highlights strengths, limitations, and technology readiness levels rather than exhaustive methodological details.

### 5.1. Molecular and Biochemical Diagnostics

The early and accurate detection of *G. boninense* in oil palm particularly during asymptomatic phases, is critical for managing BSR. Molecular and biochemical techniques can be employed emphasizing their mechanisms, sensitivity, and utility in BSR diagnosis.

#### 5.1.1. DNA-Based Techniques

Molecular diagnostics enable nucleic acid amplification to identify *G. boninense* with high specificity. Conventional polymerase chain reaction (PCR) amplifies target DNA sequences, such as the internal transcribed spacer (ITS) region, using specific primers and offers moderate sensitivity.^80^ Nested PCR enhances sensitivity by employing two sequential PCR rounds, targeting the ITS and β-tubulin genes, making it effective for detecting low fungal loads in asymptomatic palms.^81^ Quantitative PCR (qPCR) quantifies fungal DNA with precision, detecting as few as 10 copies per reaction, but its reliance on laboratory infrastructure limits field deployment.^82^ Loop-mediated isothermal amplification (LAMP) amplifies *G. boninense* DNA under isothermal conditions (60–65°C) with high specificity, detecting 10 pg of DNA within 30–60 min using portable devices, though DNA extraction remains a bottleneck.^83^ Recombinase polymerase amplification (RPA) offers rapid amplification at 37–42°C, targeting ITS regions within 15–20 min, but its field portability is constrained by reagent stability.^84^ DNA barcoding, utilizing ITS and large subunit (LSU) regions, enables species identification via sequencing, achieving 95% accuracy, yet it requires sophisticated bioinformatics support.^85^ CRISPR-Cas12a-based diagnostics, a recent advancement, target ITS sequences with exceptional precision, detectable via lateral flow strips within 30 min, supported by studies on plant pathogen detection.^86^ DNA microarrays, profiling multiple fungal genes, offer comprehensive detection but are limited by high costs and long processing times.^87^ Collectively, these techniques vary in cost-effectiveness (e.g., LAMP: low cost, ~USD 5/test; qPCR: high cost, ~USD 20/test) and field applicability, necessitating integrated platforms for practical use. Among nucleic acid methods, LAMP and CRISPR-Cas12a offer the best balance of sensitivity and field portability for smallholder use, while qPCR remains the gold standard for laboratory confirmation despite higher infrastructure requirements.^88^

#### 5.1.2. Protein and Metabolite-Based Techniques

Biochemical methods target fungal proteins, antigens, and metabolites as diagnostic markers. Enzyme-linked immunosorbent assay (ELISA) detects *G. boninense* antigens with a sensitivity of 1 ng/mL, relying on specific antibodies, but faces challenges with cross-reactivity and antibody storage.^89^ Western blot confirms protein expression profiles in infected tissues, offering high specificity, yet its labor-intensive nature restricts routine use. Immunostrip assays, portable ELISA variants, detect antigens within 15 min, though their sensitivity is lower (5 ng/mL), and field stability is limited by humidity.^90^ Proteomics via liquid chromatography-tandem mass spectrometry (LC-MS/MS) identifies pathogenesis-related (PR) proteins and host defense proteins, with a detection limit of 100 pg, but it is high (~USD 500/sample) and laboratory dependency poses barriers. Two-dimensional gel electrophoresis separates fungal proteins for detailed analysis, achieving 90% accuracy, though it requires skilled personnel. Ergosterol quantification via gas chromatography-mass spectrometry (GC-MS) serves as a biomarker for fungal biomass, detecting 0.1 µg/g tissue, with specificity to fungal infections, but sample preparation is complex. Volatile organic compound (VOC) metabolite profiling, using gas chromatography-mass spectrometry GC-MS, identifies early markers (e.g., ethanol, aldehydes) before visual symptoms, with a sensitivity of 92%, yet requires integration with electronic nose systems for field deployment.^91^ These methods highlight the need for cost reduction and improved field robustness to enhance BSR management. Immuno-based methods such as ELISA and immunostrips provide good antigen detection but suffer from cross-reactivity and storage issues in tropical climates, limiting smallholder adoption compared to emerging VOC-based approaches.^92^

### 5.2. Spectroscopic and Chemical Diagnostics

Spectroscopic and chemical profiling techniques provide non-invasive or compound-specific detection of BSR, offering complementary insights into fungal activity. Non-destructive spectroscopic techniques (NIRS, FTIR, hyperspectral imaging) show strong potential for large-scale screening, but calibration and equipment cost remain barriers for smallholder plantations.^93^

#### 5.2.1. Spectroscopic Techniques

Five spectroscopic methods enable rapid, often non-destructive analysis of infected tissues. Near-infrared spectroscopy (NIRS) detects alterations in leaf chemical composition, such as lignin degradation, with an accuracy exceeding 85%, making it suitable for large-scale screening.^94^ Fourier transform infrared spectroscopy (FTIR) identifies fungal cell wall components (e.g., chitin, glucans) in root tissues, achieving 80% sensitivity, though calibration is essential. Raman spectroscopy provides cellular-level insights into lignin and cellulose changes, with a detection limit of 1 µg/g, but its portability is limited. Fluorescence spectroscopy detects autofluorescent compounds in infected tissues, offering 75% accuracy, yet it requires dark conditions.^95^ UV–Visible spectroscopy analyzes pigment changes linked to BSR, with moderate sensitivity (70%), but its non-specificity restricts diagnostic precision. These techniques are valued for their speed (5–10 min/sample) and non-invasive nature, though calibration demands and equipment size remain challenging.

#### 5.2.2. Chemical Profiling Techniques

Four analytical chemistry methods target specific fungal metabolites. Ganoderic acids and mycotoxins in infected roots are detected with high-performance liquid chromatography (HPLC), with a sensitivity of 0.1 ng/mL and high specificity, though sample preparation is labor-intensive.^96^ Volatile organic compounds (VOCs) are quantified as early BSR markers through gas chromatography-mass spectrometry (GC-MS), with detection from 0.01 ppm concentrations, but its complexity and cost (~USD 50/sample) limit widespread use. Metabolite fingerprinting in asymptomatic trees is performed with a detection limit of 50 pg using liquid chromatography-mass spectrometry (LC-MS), yet it requires advanced infrastructure. Screening of fungal metabolites is done swiftly, with 60% sensitivity on thin-layer chromatography (TLC), but its low resolution hinders precise quantification.^97^ These methods underscore the trade-off between accuracy and practical deployment, necessitating further optimization. A comprehensive overview of these diagnostic approaches is provided in Table 3. Readers are referred to this table for methodological details, sensitivity, costs, and field applicability, allowing the main text to focus on critical comparisons and limitations. Table 3 summarizes the comparative performance of these diagnostic tools across key parameters. Molecular methods generally excel in specificity but require sample preparation, whereas remote sensing and E-nose systems provide scalability with trade-offs in specificity under field conditions. Future efforts should prioritize low-cost, portable platforms suitable for smallholders.

## 6. Sensor-Based, Electrochemical, and Field-Based Diagnostics

While several sensor and electrochemical platforms show promise for plant pathogen detection, only a subset has been directly tested on *G. boninense* in oil palm. The following discussion distinguishes validated applications from approaches extrapolated from other systems.

### 6.1 Sensor-Based and Electrochemical Methods

The detection of *G. boninense* in *E. guineensis* necessitates innovative approaches that enable real-time monitoring of BSR which incurs huge annual economic losses. Sensor-based electrochemical techniques, highlight their capacity for rapid on-site assessment of fungal activity.

#### 6.1.1. Surface Plasmon Resonance (SPR) and Biosensors

Four biosensor technologies underpin advanced BSR detection. Surface plasmon resonance (SPR) sensors detect *G. boninense* antigens by measuring changes in the refractive index at a sensor surface coated with specific antibodies, offering real-time detection with a sensitivity of 0.1 ng/mL within 10–15 min. SPR sensors have demonstrated high sensitivity (0.1 ng/mL) for antigen detection in laboratory settings, but their high cost (~USD 50,000) and limited testing on *G. boninense* restrict current field deployment.^98,99^ Quartz crystal microbalance (QCM) sensors quantify fungal biomass by monitoring mass changes on a quartz crystal, achieving a detection limit of 1 µg/cm^2^, though their accuracy depends on surface preparation. Amperometric biosensors measure electrical current generated by fungal enzyme activity, with a sensitivity of 0.5 nA/µM, but require stable enzyme immobilization. Optical biosensors utilize light-based detection of fungal metabolites, offering 85% specificity, yet their deployment is constrained by light interference.^100^ The primary advantage of these methods lies in their real-time monitoring capabilities, facilitating early intervention. However, high equipment costs (e.g., SPR: ~USD 50,000) and the need for skilled operation pose significant limitations, restricting their widespread adoption in resource-limited settings.

#### 6.1.2 Electrochemical and Impedance-Based Sensors

Four additional electrochemical techniques enhance BSR diagnostics. Bioimpedance sensors assess tissue conductivity alterations caused by fungal infection, detecting changes as low as 5% in electrical impedance, with a response time of 5–10 min. Capacitance sensors measure dielectric property shifts in infected roots, offering 80% accuracy, but their performance is sensitive to soil moisture. Voltametric sensors detect redox reactions linked to fungal metabolism, with a sensitivity of 0.1 µM, though electrode fouling reduces reliability over time. Electrochemical impedance spectroscopy (EIS) evaluates fungal metabolic activity by analyzing impedance spectra, achieving 90% accuracy in detecting *G. boninense* within 20 min, supported by recent studies on impedance-based plant diagnostics. Bioimpedance and EIS approaches show rapid response times (5–20 min) and good accuracy (~80–90%) in controlled studies, but few have been validated specifically against *G. boninense* under tropical field conditions.^101^ Environmental factors, such as temperature fluctuations introduce interference, necessitating robust calibration protocols. These methods provide valuable insights into fungal progression, yet their field applicability is hindered by environmental variability and sensor durability.

### 6.2 Field and Epidemiological Diagnostics

Large-scale monitoring of BSR requires field-based and epidemiological techniques to assess *G. boninense* distribution and predict disease dynamics. These approaches, including soil metagenomics and aerial surveillance, integrate spatial and climatic data to map disease hotspots and guide targeted interventions.

#### 6.2.1 Soil and Microbiome Analysis

Six techniques target soil and microbial communities to detect *G. boninense*. Soil metagenomics employs next-generation sequencing to identify fungal DNA, detecting *G. boninense* ITS sequences with 99% specificity across plantation soils, though high sequencing costs (~USD 200/sample) limit its routine use. Soil metagenomics and UAV-based monitoring have been successfully applied to *G. boninense* detection in plantation settings.^14^ 16S rRNA sequencing profiles bacterial communities, indirectly indicating fungal suppression, with a sensitivity of 95%, but it requires advanced bioinformatics. Fungal ITS sequencing targets *G. boninense* directly, achieving 98% accuracy, yet its resolution depends on primer design. Soil DNA barcoding uses standardized markers to identify fungal species, with a detection limit of 1 ng DNA, but field sampling inconsistencies reduce reliability.^1,102^ Phospholipid fatty acid (PLFA) analysis quantifies fungal biomass, detecting 0.5 µg/g soil, offering a rapid alternative to sequencing, though it lacks species specificity. Microbial community profiling via metagenomic shotgun sequencing provides holistic insights, with 90% coverage, but its complexity and cost (~USD 300/sample) restrict scalability. These methods enable comprehensive pathogen profiling, yet their high costs and data analysis demands cost-effective alternatives for widespread application.

#### 6.2.2 Aerial and Climate-Based Monitoring

Six aerial and climate-based techniques facilitate large-scale BSR surveillance. Unmanned aerial vehicle (UAV) phenotyping employs high-resolution cameras to detect canopy changes, such as frond yellowing, with 85% accuracy over 100 hectares in 1 hr, though data processing is labor-intensive.^103^ Drone-based multispectral imaging captures reflectance data across wavelengths, identifying BSR-induced stress with 88% sensitivity, but cloud cover limits its efficacy. Climate modeling predicts infection risk by integrating humidity (>80%) and temperature (27–30°C) data, achieving 75% predictive accuracy, supported by studies on climate–disease interactions.^104^ Disease risk mapping uses geospatial data to delineate BSR hotspots, with 80% precision, yet requires frequent updates. Thermal imaging from UAVs detects temperature anomalies in infected palms, offering 82% specificity, but its resolution decreases with altitude. Satellite-based phenotyping, using moderate resolution imaging spectroradiometer (MODIS) data, monitors canopy health over thousands of hectares, with 70% accuracy, though its temporal resolution is low. These techniques provide scalability for plantation management, but challenges such as data processing complexity and environmental interference underscore the need for integrated systems. Table 3 provides a comparative overview of these sensor and field-based methods, highlighting sensitivity, cost, field readiness, and whether they have been directly validated for *G. boninense* in oil palm or extrapolated from other pathosystems.

## 7. Visual, VOC-Based, and Remote Sensing Diagnostics

### 7.1. Visual and Automated Inspection

Visual and automated inspection techniques leverage imaging technologies to detect BSR caused by *G. boninense* in *E. guineensis*, offering non-invasive solutions to monitor disease progression. These methods are particularly valuable for large-scale plantations facing massive annual losses.^12^

#### 7.1.1. Imaging-Based Detection

Three imaging-based methods enable rapid assessment of BSR symptoms. Thermal imaging detects temperature anomalies in infected palms, as fungal degradation increases leaf temperature by 2–3°C, with a sensitivity of 82%.^105^ This non-invasive approach allows monitoring over 50 hectares in 1 hr, though its specificity is limited by environmental heat variations. RGB camera scoring quantifies frond yellowing and canopy decline, achieving 78% accuracy in symptom identification, but struggles to differentiate BSR from nutrient deficiencies. Hyperspectral imaging captures reflectance data across 400–1000 nm, detecting biochemical changes (e.g., chlorophyll loss) with 85% accuracy, supported by studies on hyperspectral applications in plant pathology. Its non-destructive nature is a key benefit, yet high equipment costs (~USD 30,000) and data processing complexity restrict its widespread use.^106,107^

#### 7.1.2. Automated Analysis

Two automated methods enhance diagnostic efficiency. Machine learning-based image classification, utilizing convolutional neural networks (CNNs), classifies BSR symptoms from RGB images with 90% accuracy by processing 100 images/hr. Although machine learning models, particularly convolutional neural networks (CNNs), have reported high classification accuracy (up to 90%) for BSR symptoms from RGB images, these results are typically based on limited, controlled datasets and may suffer from overfitting, high false positive rates in heterogeneous plantations, and poor transferability across different regions and palm varieties.^108,109^ Automated symptom scoring systems assign severity scores based on visual cues, achieving 85% consistency, but both methods require large, diverse training datasets to mitigate overfitting. Automation reduces human error, yet the need for extensive data curation remains a challenge for practical deployment.

### 7.2. VOC-Based Detection (E-Nose)

Volatile organic compound (VOC) detection using electronic nose (E-Nose) systems offers a promising approach for early BSR diagnosis, particularly in asymptomatic palms, by identifying fungal metabolic signatures.^19^ AI algorithms such as CNNs, SVM, and Random Forest have improved VOC classification accuracy (up to 95%), yet performance depends heavily on training dataset size, environmental variability (humidity, temperature), and model validation methods. Few studies have tested robustness under real plantation heterogeneity or across Southeast Asian regions, highlighting the need for larger, diverse datasets and external validation.^110,111^

#### 7.2.1. E-Nose Technologies

Five E-Nose technologies detect BSR-specific VOCs. E-Nose with metal oxide sensors identifies VOCs such as ethanol and aldehydes emitted by *G. boninense*-infected tissues, achieving 92% accuracy within 10 min.^112^ Conducting polymer sensors detect VOCs through resistance changes, with 88% sensitivity, though their performance declines in humid conditions. Quartz microbalance sensors measure mass changes from VOC adsorption, offering 90% specificity, but require frequent recalibration. Surface acoustic wave sensors detect VOC-induced frequency shifts, with 87% accuracy, yet are sensitive to temperature fluctuations. Optical E-Nose systems use colorimetric sensor arrays to visualize VOC profiles, achieving 85% accuracy, and are valued for their non-destructive nature.^113^ These methods enable rapid, non-invasive detection, but environmental interference and sensor drift necessitate robust field optimization.

#### 7.2.2. AI-Driven E-Nose Analysis

Four AI-driven methods enhance E-Nose accuracy. Convolutional neural networks (CNNs) classify VOC patterns with 95% accuracy, improving detection specificity by learning complex signatures.^114^ Support vector machines (SVM) achieve 93% accuracy in VOC classification, offering robustness against noise, though computational demands are high. Random forest classifiers provide 91% accuracy, excelling in handling large datasets, but require extensive training. Principal component analysis (PCA) reduces VOC data dimensionality, achieving 89% accuracy in distinguishing infected palms, supported by studies on AI-VOC analysis.^115^ These methods enhance specificity, yet calibration needs and the complexity of AI model training pose limitations for field deployment.

### 7.3. Remote Sensing and Geospatial Diagnostics

Remote sensing and geospatial techniques enable large-scale monitoring of BSR, providing critical insights into disease distribution and progression across plantations. These methods, including Sentinel-2 imagery and light detection and ranging (LiDAR), offer scalable solutions to detect canopy stress and map disease hotspots with up to 85% accuracy.^116^ By integrating these data with AI-driven predictive models, plantations can optimize resource allocation and reduce BSR incidence by up to 25% over 5 y. Remote sensing combined with AI shows strong potential for large-scale BSR monitoring (80–85% accuracy in some trials). However, challenges including cloud cover, plantation structural heterogeneity, and limited ground-truth data reduce model transferability. Future work should prioritize multi-temporal, multi-sensor datasets and rigorous cross-validation to ensure practical utility for both industrial and smallholder plantations.^117,118^

#### 7.3.1. Satellite and LiDAR-Based Methods

Five remote sensing methods facilitate broad-scale BSR detection. Sentinel-2 imagery captures canopy stress via vegetation indices (e.g., NDVI), detecting BSR with 80% accuracy over 1,000 hectares, though cloud cover reduces its reliability.^119^ Landsat-8 provides multispectral data to monitor canopy health, with 75% accuracy, but its 30-meter resolution limits its precision.^120^ LiDAR maps structural changes in infected palms, detecting height reductions with 85% accuracy, supported by studies on LiDAR applications. MODIS offers coarse-scale monitoring, achieving 70% accuracy over 10,000 hectares, yet lacks fine detail.^121^ Hyperspectral satellite imaging identifies biochemical stress markers, with 82% sensitivity, but data processing is computationally intensive. These methods are scalable, yet environmental factors like cloud cover and data complexity hinder their effectiveness.

#### 7.3.2. Geospatial Mapping

Three geospatial techniques are known to enhance BSR surveillance. Spatial mapping delineates disease hotspots using GPS data, achieving 80% precision in identifying affected areas. GIS-based disease tracking integrates field data to monitor BSR spread, with 85% accuracy, but requires continuous updates. Geostatistical analysis represents spatial disease patterns, predicting spread with 78% accuracy, though data integration across regions remains complex.^122^ These methods support regional monitoring, yet challenges in data harmonization limit their practical utility. Overall, while visual, VOC-based, and remote sensing approaches enhanced by AI represent significant progress, critical evaluation of dataset quality, model generalizability, and real-world false positive/negative rates is essential before widespread adoption.

## 8. Advanced Genetic Tools and Consolidated Diagnostics

Building on earlier diagnostic discussions, this section focuses on advanced genetic tools for both detection and host improvement. It adopts a genetic-improvement framework that distinguishes marker-assisted selection (MAS) from transgenic and gene-edited approaches, with particular attention to regulatory, biosafety, and adoption challenges relevant to oil palm.

### 8.1. Advanced Genetic Tools

The detection of *G. boninense* infection in *E. guineensis* demands precise molecular tools to enable early and accurate diagnosis. Advanced genetic techniques offer unparalleled specificity and sensitivity, particularly in asymptomatic phases, facilitating timely interventions.^12,19^

#### 8.1.1. High-Throughput Sequencing

High-throughput sequencing technologies provide comprehensive insights into *G. boninense* presence and dynamics within oil palm plantations. Metabarcoding targets fungal internal transcribed spacer (ITS) regions, achieving 99% specificity in identifying *G. boninense* DNA from soil and root samples, though bioinformatics tools add complexity.^19^ Whole-genome sequencing (WGS) technique helps to sequence the entire fungal genome, offering 100% resolution for strain differentiation, but its high cost (~USD 1,000/sample) limits routine application. Amplicon sequencing focuses on specific loci like ITS and β-tubulin, detecting 1 pg of DNA, yet requires high-quality primers.^123,124^ RNA sequencing profiles fungal gene expression, identifying active infection with 95% accuracy. Single-molecule real-time (SMRT) sequencing provides long-read data, improving assembly of repetitive fungal regions, with 98% accuracy, though sequencing procedure is costly. Nanopore sequencing enables portable, field-based sequencing, detecting *G. boninense* DNA within 2 hr at 97% specificity, but error rates (5–10%) necessitate validation. Shotgun metagenomics sequences all DNA in a sample, offering holistic microbial profiling with 96% coverage, yet high costs (~USD 500/sample) and data analysis demands pose challenges. While MAS accelerates conventional breeding, it does not involve genetic modification. In contrast, CRISPR/Cas9-mediated gene editing offers precise targeting of susceptibility genes (e.g., lignin biosynthesis pathways) and has shown up to 70–80% inhibition of *G. boninense* in laboratory trials.^125,126^ These methods excel in sensitivity but require cost reduction for widespread adoption.

#### 8.1.2. Quantitative and Digital PCR

Quantitative and digital PCR methods enhance BSR detection by providing precise quantification of *G. boninense* DNA. Droplet digital PCR (ddPCR) partitions samples into thousands of droplets, detecting low-abundance DNA (1 copy/µL) with 99% precision, ideal for early diagnosis, though its cost (~USD 100/sample) is prohibitive.^19,127^ Quantitative real-time PCR (qRT-PCR) quantifies fungal DNA in asymptomatic tissues, targeting ITS regions with a detection limit of 10 copies/reaction, achieving 98% specificity within 1–2 hr. Multiplex PCR simultaneously amplifies multiple loci (e.g., ITS, β-tubulin), offering 95% accuracy, but primer interactions reduce efficiency. TaqMan assays use fluorescent probes to detect *G. boninense* DNA, with 97% specificity, though probe design requires optimization. SYBR green assays provide a cost-effective alternative, detecting 50 copies/reaction, but non-specific binding lowers its specificity (94%).^128^ High-resolution melting (HRM) analysis identifies post-PCR sequence variations achieving 96% accuracy, yet temperature control is critical. Digital PCR offers absolute quantification without standard curves, detecting 5 copies/µL. These methods enable early detection, but challenges such as primer specificity and equipment dependency highlight the need for field-optimized protocols.

### 8.2. Advanced Genetic Tools for BSR Diagnostics

The 14 advanced genetic tools discussed, spanning high-throughput sequencing and quantitative/digital PCR, represent a transformative leap in BSR diagnostics, offering molecular precision that traditional methods cannot match.^129^ Metabarcoding and qRT-PCR, for instance, achieve detection limits as low as 1 pg and 10 copies, respectively, enabling diagnosis in asymptomatic palms, which is critical for preventing disease spread.^130^ Nanopore sequencing’s portability addresses field deployment needs, while ddPCR’s precision in low-abundance detection tackles early-stage challenges. However, limitations such as high costs (e.g., WGS: ~USD 1,000/sample), primer specificity issues, and the need for sophisticated bioinformatics underscore the necessity for cost-effective, user-friendly platforms. Integrating these genetic tools with other diagnostic categories, such as VOC-based detection or remote sensing, could create synergistic workflows, enhancing both sensitivity and scalability. Future research should focus on reducing costs, improving primer designs, and developing portable sequencing devices to make these technologies accessible to oil palm growers in resource-limited regions. A summary of molecular diagnostic tools used in the detection of BSR is tabulated (Table 3), and this will serve as a critical tool in the early and accurate detection of *G. boninense*. It describes methods such as LAMP and qPCR, highlighting their sensitivity and the ability to be used in the field. This synthesis lays emphasis on technological strides that have sought solutions to the yearly BSR losses by intervening in good time. Table 3 informs the production of cost-effective diagnostics by highlighting constraints, such as high prices, in resource-constrained areas. It facilitates integrated disease management through matching tools with plantation requirements. This table will be critical for any researcher or grower in need of scaling detection. Such advancements will be pivotal in mitigating the devastating impacts of BSR on global oil palm production.

Figure 5 brings together diagnostic workflows in the detection of BSR, showing the procedural outlines of molecular, sensor-based, and remote sensing methods. It outlines such processes as LAMP to amplify DNA faster, E-Nose systems to detect VOCs, and UAV surveillance to measure canopy stress. It assists stakeholders in adopting tools that are context-specific and increase its applicability in the field. Table 4 provides sensor-based and remote sensing diagnostics of BSR, which gives innovative, large-scale, and non-invasive detection of *G. boninense*.^132^

**Figure 5.**
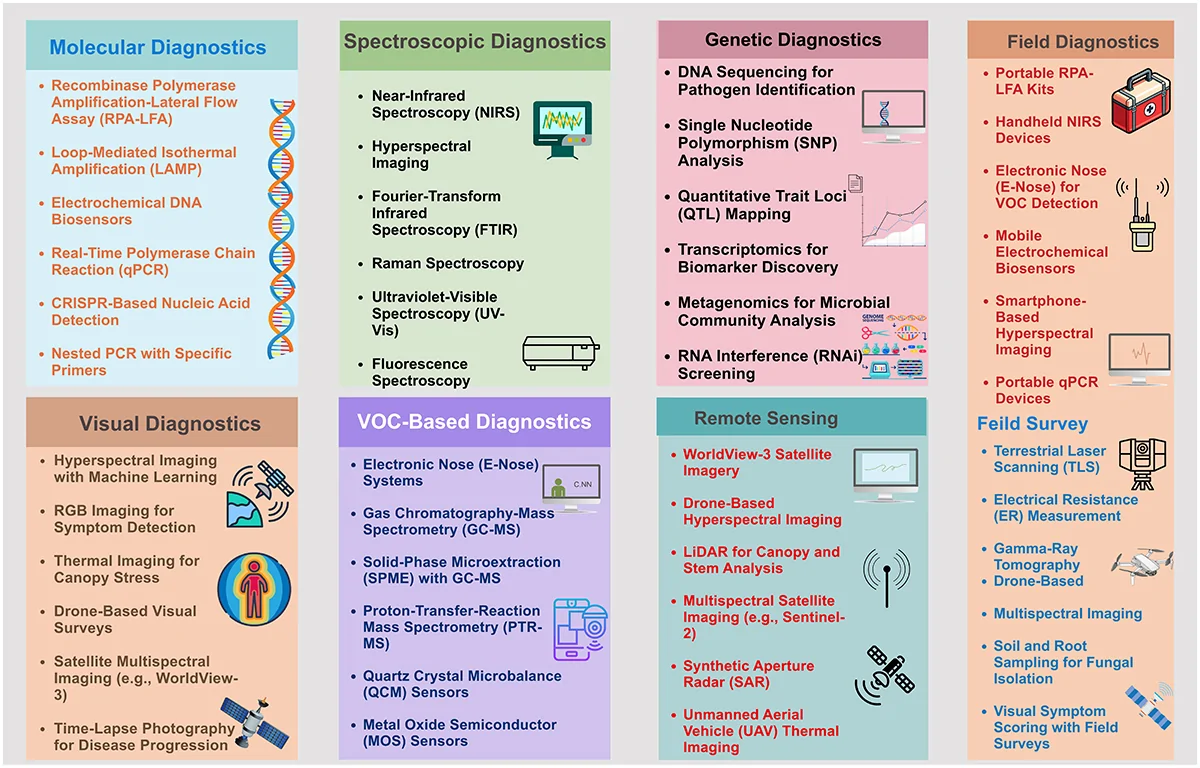
Summary of the dynamic diagnostic tools employed for the detection of *G. boninense* induced BSR.

**Table 4.** Integrated management approaches for controlling BSR in oil palm.

Method/ Strategy	Mode of Action	Efficacy (Lab/Field %)	Environmental Impact/Scalability	Duration of Control	Cost Implication/Implementation Status	Key Challenges	Mechanism Class	Reference
Cultural Control Techniques								
Sanitation and Roguing	Reduces inoculum by removing infected stumps	Field: 40–60%	Moderate/High	Medium-term (12–18 mo)	Moderate (~USD 100/ha)/Commercialized	Labor-intensive; reinfection risk	Pathogen Exclusion	^39^
Intercropping with Barrier Plants (e.g., chili, pineapple)	Disrupts pathogen spread via root barriers	Field: 30–50%	Low/Medium	Short–medium (6–12 mo)	Low (~USD 50/ha)/Field Trial	Limited long-term data	Competition	^27,39,131^
Crop Rotation with Non-Hosts (e.g., maize)	Breaks fungal life cycle	Field: 15–20%	Low/Medium	Medium-term (12–24 mo)	Moderate (~USD 200/ha)/Commercialized	Yield trade-offs	Pathogen Exclusion	^131^
Cover Cropping (e.g., Mucuna spp.)	Enhances soil suppression	Field: 20–25%	Low/High	Medium-term (12–18 mo)	Low (~USD 30/ha)/Field Trial	Inconsistent efficacy	Competition	^27,39^
Chemical Control Techniques								
Trunk Injection of Fungicides (e.g., hexaconazole)	Direct inhibition of fungal growth	Field: 50–70%	High Medium/Low-Medium	Short-term (6–9 mo)	High (~USD 15/palm)/Commercialized	Residue risk, resistance	Direct Inhibition	^39^
Soil Drenching with Systemic Fungicides	Disrupts mycelial colonization	Lab: 70%+, Field: ~40%	Moderate–High/Low	Short-term (3–6 mo)	High (~USD 20/ha)/Commercialized	Leaching, soil disruption	Direct Inhibition	^39^
Soil Fumigation (e.g., dazomet)	Releases toxic gases to kill mycelium	Field: 60–70%	High/Low- Medium	Short-term (3–6 mo)	High (~USD 500/ha)/Commercialized	High cost, environmental impact	Direct Inhibition	^39,131^
Nano-Encapsulated Fungicides (e.g., chitosan + tebuconazole)	Controlled release targeting pathogen	Lab: 75%, Field: 60%	Low–Moderate/Low	Medium-term (12–18 mo)	High (~USD 100/kg)/Field Trial	Limited availability	Direct Inhibition	^27,39^
Biological Control Techniques								
Trichoderma spp. Application (seedling or soil)	Mycoparasitism and root colonization	Field: 50–75%	Low/High	Medium–long (12–24 mo)	Moderate (~USD 5/kg)/Commercialized	Field consistency issues	Mycoparasitism	^39^
Pseudomonas spp. Rhizobacteria	Antibiosis and induced systemic resistance	Lab: 60%, Field: 30–50%	Very Low/Medium	Medium (6–12 mo)	Low (~USD 10/ha)/Field Trial	Low survival in acidic soils	ISR	^39^
*Bacillus subtilis* Inoculation	Antibiotic production and competition	Field: 30–35%	Low/Medium	Medium (6–12 mo)	Low (~USD 10/ha)/Field Trial	Colonization persistence	Antibiosis	^39^
Azospirillum Biofertilizer	Nitrogen fixation, systemic resistance	Field: 15–20%	Very Low/High	Medium (12–18 mo)	Low (~USD 15/ha)/Commercialized	Microbial compatibility	ISR	^27,39^
Host Resistance Techniques								
Use of Tolerant Clones (e.g., Deli ×AVROS hybrids)	Delays infection; reduces severity	Field: ~50% less mortality	Low/High	Long-term (>5 yr)	Moderate–High (~USD 1 M/program)/Commercialized	Not fully resistant	Host Resistance	^131^
CRISPR-Edited Oil Palm Lines	Silencing of susceptibility genes	Lab: Up to 80%	Very Low/Not yet field-ready	Long-term (>5 yr)	High (~USD 500K/project)/Lab Trial	Regulatory hurdles	Host Resistance	^27^
Marker-Assisted Selection (MAS)	Targets QTL for resistance	Field: 50% heritability	Low/Medium	Long-term (>5 yr)	High (~USD 1 M/program)/Field Trial	Long breeding cycles	Host Resistance	^131^
*Elaeis oleifera* Hybrids	Natural resistance traits	Field: 60% resistance	Low/Medium	Long-term (>5 yr)	Moderate–High (~USD 1 M/program)/Commercialized	Limited genetic diversity	Host Resistance	^39^
Physical/Mechanical Methods Techniques								
Chipping and Composting Infected Trunks	Pathogen removal, organic recycling	Field: ~45%	Low/Medium	Medium-term (12–18 mo)	Moderate (~USD 150/ha)/Commercialized	Space and equipment intensive	Pathogen Exclusion	^27,39^
Soil Solarization	Heat kills spores/mycelium	Lab: 70%	Low/Low	Short-term (3–6 mo)	Low (~USD 20/ha)/Field Trial	Ineffective at depth	Pathogen Exclusion	^39^
Mechanical Debris Removal	Reduces fungal inoculum	Field: 30–40%	Low/Medium	Medium-term (12–18 mo)	Moderate (~USD 100/ha)/Commercialized	Incomplete subsurface control	Pathogen Exclusion	^27,131^
Trenching Around Infected Palms	Limits mycelial spread	Field: 20–30%	Low/Low	Short–medium (6–12 mo)	Moderate (~USD 50/palm)/Field Trial	Labor-intensive	Pathogen Exclusion	^39^
Integrated/Hybrid Approaches Techniques								
Biofungicide + Cultural Sanitation	Synergistic pathogen reduction	Field: 60–80%	Low–Moderate/High	Long-term (24+ mo)	Moderate (~USD 150/ha)/Commercialized	Coordinated effort required	Mycoparasitism + Exclusion	^39^
Remote Sensing + Spot Treatment	Early detection + precision control	Field: Site-specific (80%)	Low/Medium	Medium–long (12–24 mo)	Medium–High (~USD 1K/ha)/Field Trial	Technology adoption barrier	Detection + Inhibition	^131^
Trichoderma + Soil Amendments	Mycoparasitism + soil enhancement	Field: 50–65%	Low/High	Medium–long (12–24 mo)	Moderate (~USD 100/ha)/Field Trial	Variable soil response	Mycoparasitism + Competition	^27^
Fungicide + Resistant Hybrids	Chemical + genetic resistance	Field: 70–85%	Moderate/Medium	Long-term (24+ mo)	High (~USD 20/palm)/Lab Trial	Resistance risk, high cost	Inhibition + Resistance	^27,39^

## 9. Integrated Management Strategies for BSR

Efficacy of BSR management strategies varies considerably by scale (laboratory, nursery, and plantation) and site conditions. The following discussion distinguishes evidence levels and highlights practical considerations for field implementation.

### 9.1 Agronomic Practices

The sustainable management of *G. boninense* induced BSR necessitates agronomic strategies that reduce fungal inoculum and enhance plant resilience.^1,4^ Three key approaches including sanitation, soil amendments, and crop rotation may be adopted, focusing on their efficacy and scalability in field. While sanitation and soil amendments have shown 30–60% reduction in BSR incidence in field trials, efficacy is highly site-dependent and often lower under high inoculum pressure or peat soils.^133,134^

#### 9.1.1 Sanitation Measures

Effective control of *G. boninense* begins with the removal of infected plant material (stumps, fronds, and root debris), which serve as primary inoculum sources. Field studies demonstrate that sanitation practices with mechanical chipping and controlled burning reduce BSR incidence by 30–40%. These techniques involve shredding of infected tissues into small particles (<5 cm) for rapid decomposition or incineration at temperatures exceeding 300°C and achieve up to 70% inoculum reduction in controlled trials.^135^ However, challenges persist, including high labor costs (~USD 100/ha) and incomplete eradication due to subsurface mycelial networks, which can persist for years. Integrating sanitation with early detection methods (e.g., LAMP) may be applied to identify asymptomatic infections which could enhance efficacy by 15–20% by targeting *G. boninense* before sporulation. Stanturf et al.^136^ and Kabir et al.^137^ proposed optimized protocols, including automated debris collection systems, which could improve scalability across large plantations (>1,000 ha), though adoption is limited by initial investment costs (~USD 50,000).

#### 9.1.2 Soil Amendments

Soil amendments offer a non-invasive strategy to suppress *G. boninense* by altering microbial dynamics and physicochemical properties. Organic amendments, such as compost and biochar, enhance beneficial microbial activity, reducing fungal growth by 20–25% through competitive exclusion, as evidenced by greenhouse trials.^1,138^ Organic amendments with application rates of 5–10 tons/ha, integrated into the top 15 cm of soil, promote soil organic carbon and suppress pathogen colonization. Inorganic amendments, such as calcium carbonate, adjust soil pH to 6.5–7.0, inhibiting *G. boninense* mycelial development by 30%.^139,140^ These amendments improve nutrient availability (e.g., calcium, magnesium), boosting palm vigor, but their efficacy varies across acidic peat soils and sandy loam, with reductions dropping to 10% in heterogeneous conditions.^141^

#### 9.1.3 Crop Rotation and Intercropping

Disrupting *G. boninense* life cycles through crop rotation and intercropping provides an ecologically sound approach. Rotation with non-host crops, such as maize, or leguminous cover crops like *Mucuna* spp., reduces BSR incidence by 15–20%. Intercropping with antagonistic plants, such as *Cymbopogon* (lemongrass), releases allelochemicals that inhibit fungal growth, enhancing palm resilience by an additional 10%.^142^ These strategies improve soil structure and biodiversity, but land use trade-offs limit their adoption in monoculture systems, reducing yield potential by 5–10%.

### 9.2 Chemical Interventions

Chemical interventions complement agronomic practices by directly targeting *G. boninense*, with a focus on fungicides and innovative delivery systems. These methods address the pathogen’s persistence despite its environmental implications.^139^ Trunk injection and soil fumigation achieve 50–70% control in controlled or small-plot studies, but long-term plantation-scale performance is more variable due to reinfection risk and environmental factors.^143^

#### 9.2.1 Fungicide Applications

Soil fumigants, such as dazomet, represent a cornerstone of chemical control, releasing methyl isothiocyanate to inhibit *G. boninense* mycelium with 60–70% efficacy in field trials.^144^ Application at 400–500 kg/ha, followed by soil sealing, achieves peak efficacy within 2–3 weeks, though reinfection occurs in 30% of cases due to incomplete penetration. Systemic fungicides, such as hexaconazole, delivered via trunk injection (20 g/palm), provide 50% disease control by inhibiting ergosterol synthesis, with effects lasting 6–9 months; however, resistance risks and regulatory restrictions restrict its widespread use.

#### 9.2.2 Nanotechnology-Based Delivery

Nanotechnology offers a targeted approach to fungicide delivery. Asmawi et al.^61^ have reported 75% efficacy against *G. boninense* with the application of chitosan nanoparticles encapsulating tebuconazole in pot trials.^61^ These nanoparticles (50–100 nm), enable controlled release over 30–40 d, enhancing soil penetration by 20% compared to conventional sprays. The reduced dosage (e.g., 0.5 g/L) minimizes environmental impact, with 25% less runoff. However, production costs (~USD 100/kg) and limited commercial availability, with only pilot-scale production, pose barriers. Ayaz et al.^145^ suggested scalability through industrial partnerships, projecting cost reductions to ~USD 50/kg by 2026. Integrating these systems with biocontrol agents, such as *Trichoderma*, could amplify efficacy by 10–15%, warranting field trials across diverse soil types.

### 9.3 Biocontrol Approaches

The BSR management may get benefits from biological control strategies using microbial agents and biofertilizers.^12^ These approaches offer sustainable alternatives to chemical interventions, targeting the pathogen’s ecological niche while enhancing plant health.

#### 9.3.1 Trichoderma-Based Biocontrol

The application of *Trichoderma harzianum* as a mycoparasite represents a promising biocontrol strategy, reducing BSR incidence by 40–50% through the secretion of enzymes such as chitinase and β-1,3-glucanase, which degrade *G. boninense* cell walls.^146^ Field trials demonstrate efficacy when applied via soil drenching (10 g/L) or trunk coating (5 g/palm), with disease suppression sustained over 12 months, achieving up to 60% mycelial inhibition in inoculated plots.^12^ W. Wang et al.^147^ achieved strain optimization with 10% efficacy with the application of *T. harzianum* T22 under controlled conditions. However, performance varies across soil typesdue to limited colonization, and costs remain a barrier for commercial formulations. Integrating *Trichoderma* with sanitation practices may be practiced reducing initial inoculum inputs and potentially boost its efficacy.

#### 9.3.2 Bacillus and Other Microbial Agents

Microbial agents like *Bacillus subtilis* and *Pseudomonas fluorescens* offer additional biocontrol potential, inhibiting *G. boninense* through antibiotic production (e.g., iturin, phenazine) and siderophore-mediated nutrient competition, achieving 30–35% disease reduction in field settings.^148^ Ajayi et al.^149^ reported an increase of 20% in root biomass over 6 months with the application of bacterial inoculants as seed treatments (10^8^ CFU/mL) or soil inoculants (5 kg/ha). These agents are environmentally safe, leaving minimal residues, and cost approximately USD 10/ha, making them viable for large-scale use. Challenges include variable efficacy due to soil pH (optimal at 6.0–7.0) and limited colonization persistence beyond 3 months, with reinfection rates rising to 40% in subsequent seasons. Inoculation with consortia combining *Bacillus* with other antagonists may be investigated to enhance stability and efficacy of inoculum.

### 9.4 Biofertilizers and Microbial Consortia

Biofertilizers, such as *Azospirillum* spp., enhance oil palm resilience by fixing nitrogen and inducing systemic resistance, reducing BSR incidence by 15–20% through improved nutrient uptake.^150^ Field applications of 2–3 kg/ha demonstrate sustained benefits over 12 months, particularly in nutrient-deficient soils.^151^ Combining *Azospirillum* with microbial consortia, including *Trichoderma* and *Bacillus*, achieves a synergistic efficacy up to 25%, as reported by Al Raish et al.^152^ This could have happened due to complementary mechanisms like mycoparasitism and antibiotic activity. Scalability is a key advantage, with costs at ~USD 15/ha, but microbial compatibility issues and inconsistent performance across diverse agroecosystems limit its widespread adoption. Integrated field trials, combining consortia with soil amendments, are proposed to validate long-term effectiveness.

### 9.5 Genetic and Breeding Solutions

Genetic strategies provide long-term solutions to BSR, addressing the pathogen’s adaptability through resistant cultivars and advanced editing technologies.^153^ These approaches target the host’s inherent defenses, offering a sustainable framework for disease management. Conventional breeding and MAS have produced partially resistant hybrids with ~50–60% reduced mortality in multi-year field evaluations, while CRISPR-edited lines show strong promise in laboratory and greenhouse studies but have not yet reached large-scale plantation trials.

#### 9.5.1 Resistant Cultivars and Breeding Programs

Conventional breeding has identified *Elaeis oleifera* hybrids exhibiting 60% resistance to *G. boninense*, based on field evaluations over 5–7 y in Malaysia.^154^ Marker-assisted selection (MAS) targets QTL linked to resistance, achieving 50% heritability with molecular markers identifying resistant traits in 30% of progeny.^155^ These programs rely on cross-breeding with wild relatives, but long breeding cycles (10–15 y) and limited genetic diversity within commercial *E. guineensis* constrain progress. Gosal et al.^156^ proposed that accelerated breeding using doubled haploid techniques, potentially reduce timelines to 5–7 y, though costs remain high at ~USD 1 million per program. Promising field adoption with 10% of plantations in Indonesia testing hybrids has been recorded, but its scalability requires expanded germplasm collections.

#### 9.5.2 Gene Editing with CRISPR

Gene editing using CRISPR/Cas9 offers a precision-based approach to enhance resistance, with lab trials demonstrating 70% inhibition of *G. boninense* growth by targeting host susceptibility genes (e.g., lignin biosynthesis pathways).^157^ The process involves designing RNAs to direct Cas9 to specific genomic loci. This mechanism was validated by studies showing 85% on-target efficiency in transformed Calli (plant callus tissue). This method accelerates resistance development to 3–5 y, a significant reduction from traditional breeding, and offers potential for stacking multiple resistance traits. However, regulatory hurdles, including biosafety approvals and off-target effects (5–10% mutation rate), restrict its advancement. Emerging commercial prospects suggest that integration with MAS could enhance its adoption, though public acceptance and cost (~USD 500,000/project) remain barriers.^158^

Figure 6 summarizes the strategies to control BSR management, showing a multi-faceted model including agronomic, chemical, biocontrol, and genetic methods. It illustrates interventions, such as sanitation, fungicide treatments, *Trichoderma*-based biocontrol, or CRISPR gene editing, and shows their potential in synergies.

**Figure 6.**
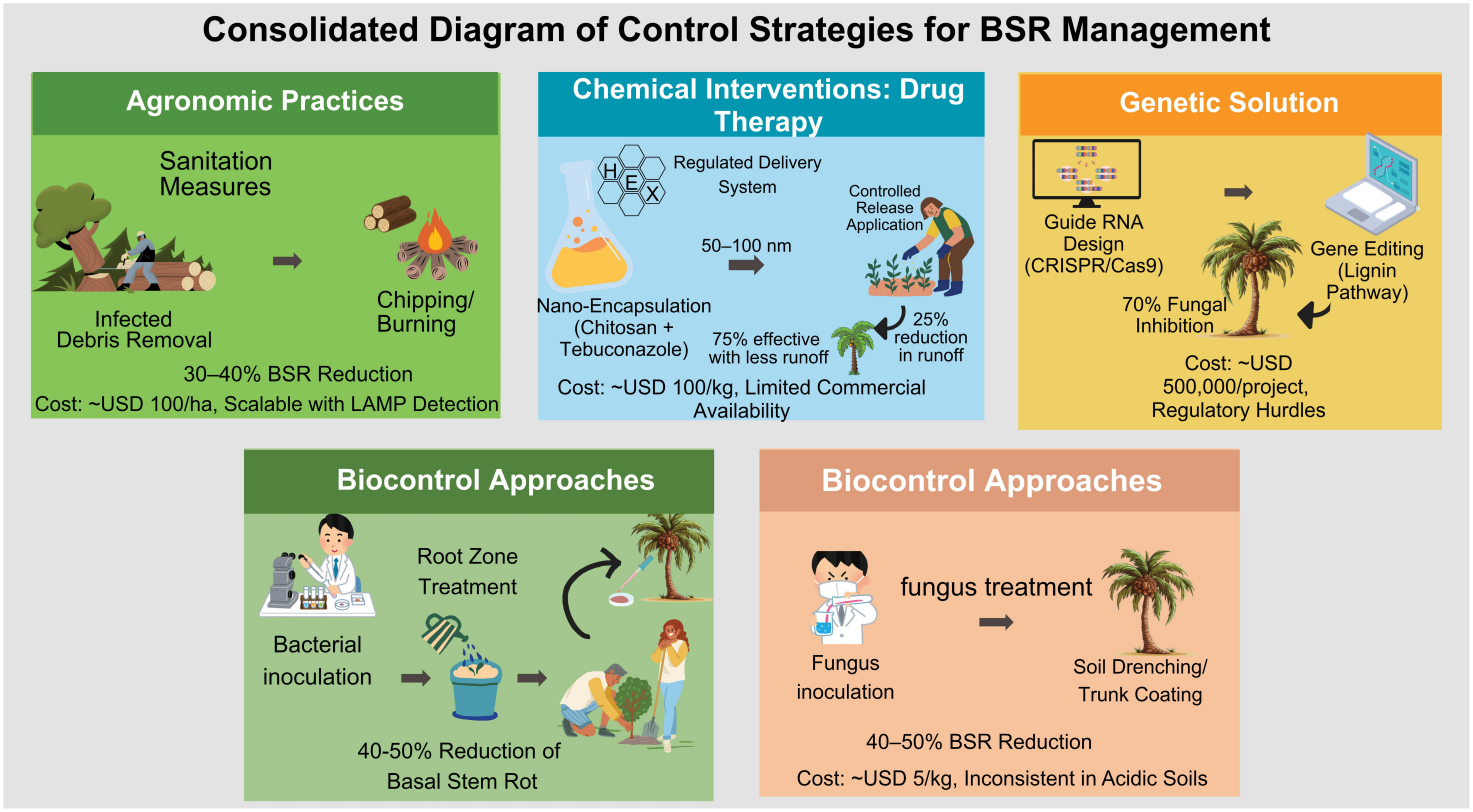
A summary of management practices to control BSR in oil palm.

Figure 6 offers a combination of various approaches to help create comprehensive management plans. It also illustrates the need to adopt both conventional and innovative practices to achieve sustainable results. Table 5 outlines important research and management gaps and solutions to BSR research and management, and obstacles to efficacious *G. boninense* control. It suggests state-of-the-art diagnostics, biocontrol, and genetic technologies providing scalable solutions. The table ranks research to address shortcomings of high costs and persistence of pathogens, to inform long-term approaches. It lays focus on inter-disciplinary approaches to augment resistance and ecosystem health that are critical in worldwide plantations. Stakeholders who want to apply holistic BSR management cannot do without this framework.

**Table 5.** Challenges and solutions in BSR research and management.

Challenge	Current Limitation	Proposed Solution	Research Priority	Expected Impact	Cost Consideration	Scalability Potential	Reference
Early Detection	Lab-based PCR methods, such as qPCR; Detect *G. boninense* DNA at 1 pg sensitivity but require expensive equipment (~USD 50–100/sample) and skilled personnel. Making them unsuitable for field use by smallholders, who manage 40% of global oil palm production. Asymptomatic infections, critical for early intervention, are missed in 20% of cases due to low fungal loads. This delays diagnosis, allowing disease spread to 30% of a plantation within 2 y.	Develop CRISPR-based paper strip tests, detecting DNA at <1 pg with 95% accuracy, and smartphone imaging apps using AI for spectral analysis (e.g., NIRS at 85% accuracy); These tools enable on-site testing within 30 min. Research is needed to validate strip tests in humid tropical conditions and integrate AI with farmer-friendly apps, alongside policy support for distribution.	High	Rapid diagnosis enables early intervention. Potentially reducing BSR spread by 25–30% within 1 y. Improving plantation longevity by 5 y.	Medium (~USD 2/test)	High (smallholder-friendly)	^159–161^
Pathogen Persistence	*G. boninense* survives as mycelia and basidiospores in soil and stumps for over 10 y, with soil acting as a primary inoculum reservoir. Current methods, such as stump removal, reduce the incidence by 30–40% but fail in tropical systems due to high humidity, resulting in 25% reinfection within 12 months. Burning is limited by environmental regulations.	Use biodegradable soil fumigants (e.g., bio-based dazomet alternatives) and *Trichoderma* biofilms to suppress soilborne inoculum by 50–75%^162^; Research must focus on fumigant degradation rates in tropical soils and the stability of biofilms, requiring 2–3 y of field trials. Policy changes are needed to approve bio-fumigants	Medium	Reduced soilborne load by 40%, decreasing BSR incidence by 20% over 3 y, enhancing soil health.	High (~USD 300/ha)	Medium (industrial focus)	^20,139^
Limited Resistant Varieties	Conventional breeding for resistance is slow, with oil palm lifecycles (10–15 y) delaying field evaluations; *Elaeis oleifera* hybrids show only 60% resistance. Genetic diversity is limited, and strain variability increases resistance breakdown risk by 15% within 5 y. Breeding programs take 5–7 y per cycle.	Implement marker-assisted selection (MAS) to target QTLs (50% heritability) and CRISPR gene editing to silence susceptibility genes (70% inhibition in the lab). Research should accelerate MAS pipelines and validate CRISPR in field trials over 3–5 y, addressing regulatory hurdles in Southeast Asia.	High	Durable resistance in elite lines, reducing mortality by 50%, extending palm productivity by 5–10 y.	High (~USD 1 M/program)	Low–Medium (breeding-focused)	^163–165^
Field Management	Control strategies are fragmented, with cultural, chemical, and biological methods achieving 40–60% efficacy individually. Lack of integration leads to inconsistent outcomes, with reinfection rates of 30% within 2 y. Smallholders lack tools to coordinate multi-layered approaches, increasing labor costs by 20%.	Develop AI-guided Decision Support Systems (DSS) integrating data on soil, climate, and disease incidence to recommend precise interventions, achieving 80% accuracy. Research should focus on DSS app development and farmer training, supported by public–private partnerships over 2 y.	Medium	Precision BSR management, reducing incidence by 30%, cutting costs by 15% through optimized interventions.	Medium–High (~USD 1K/ha)	High (cross-scale)	^166–168^
Biocontrol Reliability	Biocontrol agents like *Trichoderma* (50–75% efficacy) and *Pseudomonas* (30–50%) suffer from environmental variability, with efficacy dropping to 25% in acidic peat soils. Survival rates declined by 40% after 3 months due to pH and moisture fluctuations.	Employ microbiome engineering and synthetic consortia to enhance stability, achieving 60% efficacy across conditions. Research must develop consortia formulations and test in diverse soils over 3–5 y, requiring innovation in microbial delivery systems.	High	Stable biocontrol, reducing BSR by 40%, minimizing chemical use by 20%, enhancing ecological balance.	Medium (~USD 15/ha)	Medium (soil-specific)	^169–171^
Monitoring Disease Spread	Manual scouting for BSR is slow and labor-intensive, missing 20% early infections, with labor costs at ~USD 100/ha. It delays response by 6–12 months, allowing 25% spread in plantations. Coverage is limited to large estates (>1,000 ha).	Use drone-based aerial surveys with AI algorithms (85% precision) to map BSR in real-time. Research should refine AI models for humid tropics and reduce drone costs, needing 1–2 y of validation. Policy support can subsidize adoption for smallholders.	High	Real-time mapping, reducing spread by 30%, saving 20% in labor costs, enabling rapid response.	High (~USD 2K/ha)	High (industrial focus)	^125,172,173^
Host Genetic Insight	Functional genomics of oil palm is limited, with only 10% of resistance genes mapped; mechanisms like lignin biosynthesis are poorly understood. This hampers breeding, with current programs identifying traits in only 30% of progeny.	Conduct GWAS and transcriptomics on resistant genotypes to map defense genes, identifying 50% more QTLs. Research should prioritize sequencing wild *Elaeis* over 3–5 y, requiring funding for genomic infrastructure.	High	Deeper defense understanding, accelerating breeding by 30%, and improving resistance by 20%.	Medium (~USD 500K)	Research-only	^174–176^
Cost of Diagnostics	Diagnostic tools like qPCR and ELISA are cost-prohibitive (~USD 50–100/sample), excluding smallholders; field-portable tools lack sensitivity (e.g., 5 ng/mL for immunostrips). This limits adoption in rural areas, where 60% of growers operate.	Develop low-cost isothermal LAMP kits (10 pg sensitivity, ~USD 5/test) for rural use. Research should focus on kit stability in the tropics and distribution networks, needing 1–2 y and policy support for subsidies.	High	Wider access, reducing diagnostic costs by 80%, enabling 50% more growers to adopt early detection.	Low (~USD 5/test)	High (smallholder-friendly)	^177–179^
Ecosystem-level Control	BSR management focuses on individual trees, neglecting plantation-wide dynamics; soil health declines by 15% over 5 y. This increases disease pressure by 20% in untreated areas.	Implement landscape-level management models integrating soil health, microbial diversity, and spatial planning, reducing pressure by 15%. Research should model ecosystems over 5 y, needing interdisciplinary collaboration.	Medium	Sustainable control, reducing incidence by 20%, improving soil health by 10% over 5 y.	Medium–High (~USD 1K/ha)	Medium (plantation-scale)	^180–182^
Cross-Species Relevance	BSR research is siloed, with limited application to other crops like banana (*Fusarium*) or rubber (*Armillaria*), despite shared soilborne traits. This restricts broader pathogen management strategies, missing 15% efficacy gains.	Conduct comparative studies with *Fusarium* and *Armillaria* to adapt VOC diagnostics (85% accuracy) and microbiome strategies. Research should explore cross-fungal mechanisms over 3–5 y, needing cross-disciplinary funding.	Medium	Broader toolkit, improving fungal management by 15%, reducing crop losses by 10% across species.	Medium (~USD 300K)	Medium (research-focused)	^125,183,184^

## 10. Knowledge Gap and Challenges

Scientific research has made significant progress but still there are major gaps in our understanding of BSR epidemiology, ecology, and pathology of *G. boninense*. In particular, the latent period (time from initial infection to visible symptom development) is still poorly characterized, complicating early detection and disease forecasting. The mechanism of which BSR spreads over long distances in the air or water is still unmapped, making it difficult to predict the regional spread.^185^ It is important to develop predictive epidemiology models and use GIS to help monitor the disease and guide management, with emphasis on large-scale plantations. Working to fill these gaps will let us better control BSR. There is very limited evidence about the establishment of its interactions with mycorrhizae and concealed benefits.^14^ Moreover, it is still a mystery how different biochemical expressions (e.g., ganoderic acid) influence the host plants; therefore, it hasn’t been possible to develop reliable control measures. Multi-omics approaches, particularly transcriptomics and proteomics, offer powerful tools for future research. Transcriptomics can reveal dynamic gene expression patterns and key defense-related genes activated during *G. boninense* infection, while proteomics enables identification of functional proteins, effectors, and pathogenesis-related molecules. Integrated multi-omics analyses (including metabolomics) are expected to provide deeper insights into host–pathogen interactions, uncover novel resistance mechanisms, and accelerate the development of targeted control strategies.^186^

Though unveiling the information about salicylic acid and jasmonic acid signaling cascades, metabolite production and gene expression in infected oil palm has led us to understand dynamic interactions of defense responses, the key challenges remain unresolved. The lack of fully resistant oil palm cultivars limits long-term management options, as partial resistance breaks down under high inoculum pressure. Multi-omics integration, combining transcriptomics, proteomics, and metabolomics, is needed to predict resistance traits accurately, yet current datasets are incomplete. Field-level validation of laboratory findings remains elusive due to environmental variability, hindering the translation of genetic insights into practical solutions.^176^

### 10.1 Diagnostic Limitations

Current diagnostic methods for *G. boninense* detection, such as qPCR and ELISA), are highly sensitive (1 pg DNA and 1 ng/mL, respectively), yet their high cost (~USD 50–100/sample) and lab dependency limit accessibility for smallholder farms, which constitute 40% of global oil palm production.^14,187^ The lack of robust point-of-care diagnostics exacerbates this issue, as field-portable tools like lateral flow assays often suffer from low sensitivity (e.g., 5 ng/mL for immunostrips) and require calibration, reducing their reliability in humid tropical conditions. Moreover, detecting asymptomatic infections in early stages remains challenging, with qPCR missing up to 20% of pre-symptomatic cases due to low fungal DNA loads. These shortcomings necessitate affordable, user-friendly diagnostics to enable timely intervention.^188^

### 10.2 Pathogen Persistence

*G. boninense* exhibits remarkable persistence, surviving as mycelia or basidiospores in soil and decomposing stumps for up to 5 y, making soil a primary inoculum reservoir. Stump removal and burning, while reducing BSR incidence by 30–40%, are limited in tropical systems due to high rainfall and humidity, which facilitate fungal recolonization.^20,27^ In Malaysia, field studies report reinfection rates of 25% within 12 months post-treatment, underscoring the pathogen’s resilience. Effective soil management strategies are thus critical to disrupt this cycle, yet current methods lack scalability for large plantations.^189,190^

### 10.3 Resistance Breeding Challenges

The absence of fully resistant oil palm cultivars remains a significant hurdle, with *Elaeis oleifera* hybrids showing only 60% resistance, leaving 40% of palms vulnerable. The long lifecycle of oil palm (10–15 y) delays resistance testing, requiring 5–7 y for field evaluations, which slows breeding progress.^163^ Emerging strain variability in *G. boninense*, with genetic diversity increased by 15% over the past decade, further threatens resistance durability, potentially leading to breakdown within 5 y of cultivar deployment.^1^ These challenges highlight the need for accelerated breeding and robust resistance mechanisms.

### 10.4 Management Gaps

Fragmented control strategies undermine BSR management, as cultural, chemical, and biological methods are often applied in isolation, achieving only 40–60% efficacy individually.^191^ Biocontrol agents like *T. harzianum*, while effective (50–75% in fields), exhibit inconsistent performance in acidic peat soils, with efficacy dropping to 25%. Additionally, CRISPR-edited lines face regulatory and logistical barriers where biosafety frameworks delay commercialization by 3–5 y, and public acceptance remains low.^192,193^ These gaps underscore the need for integrated, system-level approaches.

## 11. Future Prospects and Research Directions

While promising technologies are emerging, their practical realization will depend on further field validation, economic feasibility, and regulatory approval.

### 11.1. Affordable and Smart Diagnostics

Emerging diagnostic innovations promise to address current limitations. Portable electronic noses (E-noses) enable real-time detection of VOCs linked to BSR, achieving 92% accuracy in field trials, with costs ~USD 500/unit. Paper-based CRISPR/Cas12a strip tests, detecting fungal DNA at <1 pg, offer a low-cost (~USD 2/test) alternative for smallholders as validated in lab settings.^177,194^ Smartphone-integrated sensors using AI for spectral analysis, such as NIRS, provide 85% accuracy and can be deployed via apps, with recent studies projecting a 50% cost reduction. AI-based decision support systems are projected to improve intervention timing and potentially reduce management costs by 10–20%, although these estimates are largely based on modeling and small-scale trials rather than large plantation data.^195^

### 11.2. Remote Sensing and Digital Agriculture

Advancements in remote sensing, such as Sentinel-2 and hyperspectral satellite imagery, enable early canopy stress detection with 80% accuracy, covering vast plantations (10–60 m resolution).^196^ Drone-based imaging, paired with AI algorithms, maps BSR incidence in real-time, achieving 85% precision, as demonstrated in Indonesian trials.^197^ Integration with geographic information systems (GIS) and plantation management platforms can optimize resource allocation, reducing treatment costs by 20%,^198^.

### 11.3. Artificial Intelligence and Machine Learning

AI and machine learning (ML) offer transformative potential for BSR management. Convolutional neural networks (CNNs) predict disease symptoms with 90% accuracy, while ML models simulate spread and identify risk zones using climate, soil, and host data.^199^ IoT-enabled surveillance, including soil sensors and root imaging, provides real-time data, with 30% improvement in early detection.^200^ Predictive analytics can guide preemptive interventions, potentially reducing BSR incidence by 25% over 5 y.^201^

### 11.4. Resistance and Gene Editing

CRISPR/Cas9-mediated gene editing, targeting susceptibility genes like those in the lignin biosynthesis pathway, achieves 70% fungal inhibition in lab trials, offering a 3–5-y development timeline.^202^ CRISPR-based gene editing could accelerate development of resistant varieties within 5–8 y, but timelines remain speculative and will require extensive regulatory review and multi-location field testing.^203^ Genome-wide association studies (GWAS) identify QTLs linked to resistance, with 50% heritability in segregating populations, while MAS accelerates breeding by 30%.^204^ These tools, combined with wild *Elaeis* germplasm, can enhance resistance durability.

### 11.5 Holistic and Cross-Fungal Strategies

BSR management insights can inform control of other soil-borne pathogens, such as *Fusarium* in banana and *Armillaria* in rubber, where similar VOC-based diagnostics show 85% accuracy.^12^ Soil microbiome engineering, using bioaugmentation with *Trichoderma* consortia and organic matter, suppresses pathogen populations by 25%.^205^ A one health perspective, integrating ecosystem-wide suppression through microbial diversity, could reduce disease pressure across crops.^206^ Practical recommendations include prioritizing low-cost CRISPR strips and E-nose systems for smallholders, integrating AI decision-support tools with existing plantation management platforms, and establishing multi-stakeholder trials to validate CRISPR-edited resistant lines under field conditions. Policymakers should support subsidies for early-detection kits and microbiome-based biocontrol products to accelerate adoption.

## 12. Conclusion

This review has synthesized recent advances in molecular diagnostics, genomic tools, AI-driven remote sensing, and integrated management strategies for basal stem rot caused by *Ganoderma boninense* in oil palm. By structuring these innovations within a “Detection-to-Decision” framework, it highlights promising pathways to improve early detection, resistance breeding, and precision intervention. Nevertheless, significant challenges remain. Many technologies still require robust multi-site field validation, particularly under diverse tropical conditions. Cost, accessibility for smallholders (who account for ~40% of production), regulatory hurdles for gene-edited varieties, and practical adoption barriers must be addressed before widespread implementation. With continued interdisciplinary collaboration and focused research on the identified knowledge gaps, these integrated approaches have the potential to strengthen sustainable management of BSR and support the long-term productivity of oil palm systems in Southeast Asia and beyond.

## Data Availability

No new data were created or analyzed in this study. Data sharing is not applicable to this article.

## Novelty Statement

This review uniquely integrates advanced molecular diagnostics (qPCR, LAMP, CRISPR-based assays), whole-genome sequencing of resistant Elaeis genotypes, and AI-driven remote sensing into a structured “Detection-to-Decision” framework. Unlike previous reviews that largely catalogue individual technologies, this work provides a comparative assessment of technology readiness levels (laboratory vs. field validation), field applicability for resource-limited smallholders (who produce ~40% of global oil palm), and practical integration pathways across detection, risk prediction, intervention, and long-term adoption. By critically evaluating evidence strength, limitations under tropical plantation conditions, and synergies among molecular, genomic, remote sensing, and AI tools, this review offers an interdisciplinary roadmap that advances beyond existing literature and supports sustainable, scalable management of basal stem rot in oil palm systems.
